# Sialic Acid-Responsive Polymeric Interface Material: From Molecular Recognition to Macroscopic Property Switching

**DOI:** 10.1038/srep40913

**Published:** 2017-01-13

**Authors:** Yuting Xiong, Ge Jiang, Minmin Li, Guangyan Qing, Xiuling Li, Xinmiao Liang, Taolei Sun

**Affiliations:** 1 State Key Laboratory of Advanced Technology for Materials Synthesis and Processing, Wuhan University of Technology, 122 Luoshi Road, Wuhan 430070, P. R. China; 2 Key Laboratory of Separation Science for Analytical Chemistry, Dalian Institute of Chemical Physics, Chinese Academy of Sciences, 457 Zhongshan Road, Dalian 116023, P. R. China; 3 Co-innovation Center of Neuroregeneration, Nantong University, Nantong, 226019, P.R. China; 4 School of Chemistry, Chemical Engineering and Life Science, Wuhan University of Technology, 122 Luoshi Road, Wuhan 430070, P. R. China

## Abstract

Biological systems that utilize multiple weak non-covalent interactions and hierarchical assemblies to achieve various bio-functions bring much inspiration for the design of artificial biomaterials. However, it remains a big challenge to correlate underlying biomolecule interactions with macroscopic level of materials, for example, recognizing such weak interaction, further transforming it into regulating material’s macroscopic property and contributing to some new bio-applications. Here we designed a novel smart polymer based on polyacrylamide (PAM) grafted with lactose units (PAM-*g*-lactose_0.11_), and reported carbohydrate-carbohydrate interaction (CCI)-promoted macroscopic properties switching on this smart polymer surface. Detailed investigations indicated that the binding of sialic acid molecules with the grafted lactose units via the CCIs induced conformational transformation of the polymer chains, further resulted in remarkable and reversible switching in surface topography, wettability and stiffness. With these excellent recognition and response capacities towards sialic acid, the PAM-*g*-lactose_0.11_ further facilitated good selectivity, strong anti-interference and high adsorption capacity in the capture of sialylated glycopeptides (important biomarkers for cancers). This work provides some enlightenment for the development of biointerface materials with tunable property, as well as high-performance glycopeptide enrichment materials.

Creating new materials using bio-inspired strategies is one of the grand challenges for material science^1^. In fact, biological systems that interact with or adapt to varied surrounding conditions by taking advantages of cooperative multiple non-covalent interactions and hierarchical assemblies, have evolved numerous elaborate and intricate dynamic functions^2^. And, researchers have been committed to designing artificial materials to mimic these dynamic response mechanisms upon external stimuli^3^,^4^,^5^. In this regard, dynamic material systems, particularly those based on smart polymers, capable of changing their chemical or physical properties in response to subtle environment changes provide an excellent solution^6^,^7^,^8^. Remarkable advantages including excellent responsiveness, reversible conformation transformation, and synergetic interactions among functional monomers make smart polymer an ideal platform to translate the weak stimulus signals into significant changes in a variety of macroscopic properties of material^9^,^10^,^11^, which brings great potential in a broad range of bio-applications, such as biosensing^12^, drug controllable release^13^, and tissue engineering^14^.

On the other hand, sialic acids (SAs) as a class of acidic nine carbon saccharides located at the outmost end of cell surface play crucial roles in many biological processes^15^,^16^,^17^,^18^, and aberrant sialylation has been confirmed to be closely associated with cancers, particularly the liver cancer^17^,^18^. Thus, for related researches of SAs detection or sialylation profiling, it is of great importance to recognize and capture the target SA molecules, such as various free SAs, sialylated glycans, peptides or proteins^19^,^20^. Currently, commonly used approaches for SAs recognition were mainly based on phenylboronic acid derivatives or phenylboronic acid-functionalized polymer^21^,^22^, exhibiting to people a potential tool for both diagnostic and therapeutic applications related to cancers^23^. However, for some SAs derivatives, particularly sialylated glycopeptides (SGs) that were found frequently in various cancer cells and widely known as cancer biomarkers, it is still a thorny issue for researchers to achieve the specific recognition, capture, and even selective separation, due to the ultra-low abundance of the SA derivatives (lower than 0.03% in total amount of protein hydrolysates) and strong interference from other saccharides or peptides^24^,^25^. Therefore, in order to obtain comprehensive information of sialylated glycosylation sites and glycan compositions/structures from complex real biosamples, it is highly desirable to introduce unique binding force to capture SGs with excellent specificity and adequate tunability.

In biological system, carbohydrate-carbohydrate interactions (CCIs) have been revealed to play important roles in various cellular processes (Fig. 1a)^26^,^27^,^28^, e.g., cell adhesion, recognition and signaling. Generally, CCIs are considered to be weak and not capable of providing sufficient strength and specificity for the purpose of recognition toward biomolecules. But in some examples of antibody-antigen interactions based on glycoproteins, CCIs are found to be not only strong but also specific^29^. This provides an inspiration to design carbohydrate-based SA receptor based on the CCIs mechanism. However, most of researches on CCIs have long been confined to the single-molecule level, e.g., investigations of detailed characteristics^30^, model systems to study the interaction mode or quantify the energetics^31^,^32^. It remains a great challenge to extend these studies to a broader level, for example, developing a material to explore its dynamic behaviours in response to the recognition towards SA via CCIs at macroscopic level and to promote some new bio-applications.

With these considerations in mind, here we designed a novel polymer based on polyacrylamide (PAM) grafted with lactose (denoted as PAM-*g*-lactose_0.11_), as shown in Fig. 1b. In this graft-polymer, lactose functions as a SA receptor capable of recognizing and binding *N*-acetylneuraminic acid (Neu5Ac, a typical SA) via CCI, and PAM (with weight-average molecular weight of 10000) serves as the flexible polymer main chain, both of which worked cooperatively and achieved the transformation of the weak CCIs signal into the macroscopic properties (i.e. surface topography, wettability, and stiffness) switching on the polymer surface. Furthermore, the graft polymer-modified silica gels (denoted as PAM-*g*-lactose_0.11_@SiO_2_) facilitated the high-efficiency enrichment of SGs with strong anti-interference capability and high adsorption capacity.

## Results and Discussion

**Figure 2:**
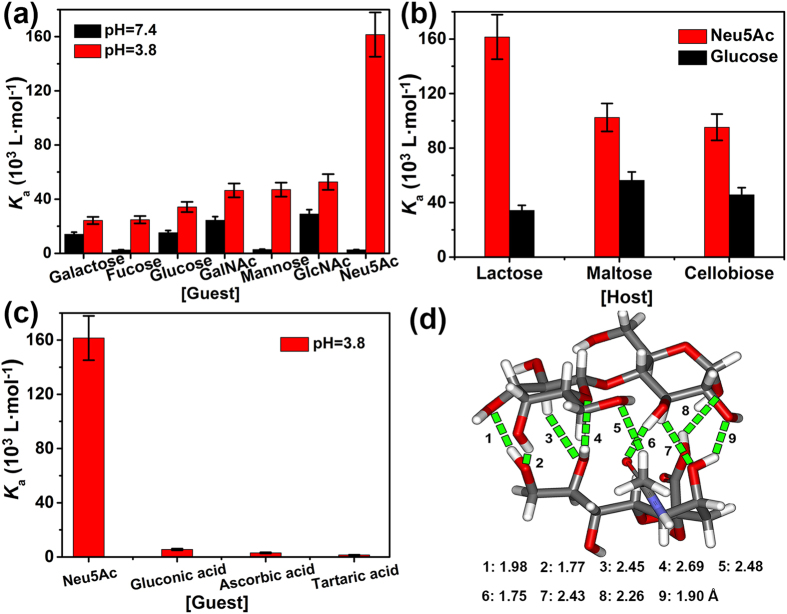
Binding affinity evaluation for screening out the optimal Neu5Ac receptor based on disaccharides. (**a**) Association constants (*K*_a_) of fluorescein-labelled lactose interacted with various monosaccharides in formate- or Tris-buffer solutions at pH 3.8 (red column) or pH 7.4 (black column), at 20 °C, *K*_a_ values were obtained from fluorescent titration experiment. (**b**) Controlled tests to screen out the optimal Neu5Ac receptor. *K*_a_ values of Neu5Ac (red column) and glucose (black column) with different disaccharide hosts in formate-buffer solutions (10 mM, pH 3.8). (**c**) Comparison of the *K*_a_ for lactose interacted with Neu5Ac and three other acidic analogues in formate-buffer solutions at pH 3.8. (**d**) The possible binding model of lactose with Neu5Ac calculated by quantum chemistry (Gaussian 2003, density functional theory at 6–311G level, H_2_O, pH 3.8), in which hydrogen bonds are depicted as green dotted lines.

Due to more hydroxyl groups with strong hydrogen bond-donating ability and more delicate molecular structures of disaccharides than those of monosaccharides, in this work, we focus on the disaccharide-based SA receptor. Firstly, the binding affinities of fluorescein-labelled lactose (a typical disaccharide) with different mono-saccharides were investigated by fluorescent titration experiment, which is a typical and reliable method for measuring the binding affinity in host-guest chemistry^33^. When the titration experiments were performed in Tris-buffer solution at pH 7.4, association constants (*K*_a_) obtained from the non-linear fitting calculation of fluorescent titration curves showed that lactose exhibited variable binding capacities towards different monosaccharides, as shown in Fig. 2a, and *K*_a_ of lactose with Neu5Ac was only 2.54 × 10^3^ L · mol^−1^. When the experiments were performed in formate-buffer solutions at pH 3.8, a remarkable increase in *K*_a_ was observed for the binding of lactose with Neu5Ac, giving a *K*_a_ value of 1.61 × 10^5^ L · mol^−1^, which is almost 64 time larger than that in neutral solution (see Supplementary Table S1), whereas no apparent change in *K*_a_ value was observed for other monosaccharides. Therefore, considering the structural features of Neu5Ac molecule, we presume the strong binding affinity of lactose with Neu5Ac in acidic condition should be closely associated with the formation of charge-reinforced hydrogen bonds between acetamido/hydroxyl groups or carboxyl groups of Neu5Ac and lactose host^34^, which deserves an in-depth investigation.

The binding affinities were also investigated using the other disaccharide-based receptors, in an attempt to identify the most favourable Neu5Ac receptor. As shown in Fig. 2b, the three tested disaccharide-based receptors, namely lactose, maltose and cellobiose, displayed much stronger binding capacities for Neu5Ac than glucose (a typical example of neutral monosaccharide) in formate-buffer solutions at pH 3.8. Among them, lactose showed the largest difference in *K*_a_ values with a *K*_a_ (Neu5Ac)/*K*_a_ (glucose) ratio of 4.73, which was considerably larger than those observed for maltose (1.82) and cellobiose (2.11). These data clearly indicated that lactose could discriminate Neu5Ac from other neutral monosaccharides more effectively. Given the fact that Neu5Ac is an acidic saccharide, control experiments for binding of lactose with three acidic analogues (i.e., gluconic acid, ascorbic acid, and tartaric acid) were performed in acidic medium (pH 3.8). As shown in Fig. 2c, results displayed that lactose still exhibited considerably strong affinity towards Neu5Ac, relative to other three acidic analogues. This implied that lactose has a pH-dependent strong binding with Neu5Ac, which could not be attributed to the acidity of Neu5Ac itself.

To further investigate the details of lactose-Neu5Ac binding, per-acetylated lactose (all hydroxyl groups were protected by acetylation) was prepared to conduct the fluorescent titration experiments. Results showed that all *K*_a_ values for per-acetylated lactose with different monosaccharides exhibited obvious decrease, compared with those for lactose with corresponding monosaccharides under the same condition (see Supplementary Table S2). This indicated that hydroxyl groups of lactose contributed to the intensive binding of lactose with Neu5Ac. Moreover, in hydrogen nuclear magnetic resonance (^1^H NMR) spectra of lactose-Neu5Ac mixture in D_2_O or DMSO-*d*_6_, the chemical shift changes for several C-H and OH protons of lactose further proved that multiple hydrogen bonds were responsible for the lactose-Neu5Ac complexation (see Supplementary Figs S1 and S2). Furthermore, this strong complexation was described by a possible binding model obtained from quantum chemistry calculation (Fig. 2d). In this model, most of hydroxyl groups in lactose participate in the binding with Neu5Ac by taking advantage of nine sets of strong hydrogen bonds. In addition, it has been proved that the addition of high concentration of urea or thiourea will destroy hydrogen bonding interaction in host-guest chemistry^35^. To further investigate the influence of urea on the complexation of lactose with Neu5Ac, we performed a control titration experiment that urea (20-fold quantity of lactose host) was added into the mixture solution. Under this condition, no evidential fluorescent intensity change (lower than 1%) was observed upon the addition of different amounts of Neu5Ac guest (see Supplementary Fig. S3). This implied a relative weak binding between lactose and Neu5Ac in the presence of urea, which further confirmed the nature of hydrogen bonding interaction between lactose and Neu5Ac.

**Figure 3:**
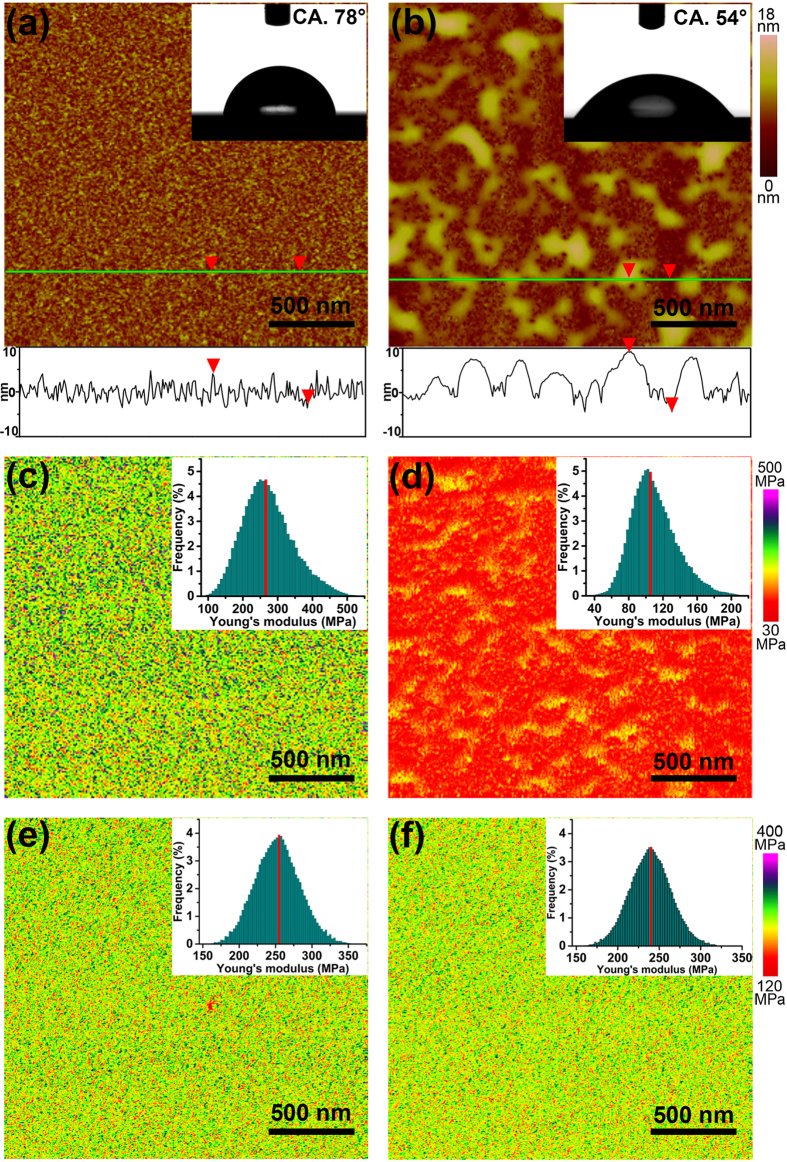
Remarkable surface topography and stiffness change induced by Neu5Ac adsorption. Surface topography (**a**,**b**) and Young’s modulus images (**c**,**d**) of PAM-*g*-lactose_0.11_ film before (**a**,**c**) and after (**b**,**d**) being treated by a Neu5Ac solution with a concentration of 0.02 mol · L^−1^ and a pH value of about 3.0. (**e**,**f**) Young’s modulus images or PAM-*g*-lactose_0.11_ film after being treated by the galactose solution (**e**) and glucose (**f**) solution, respectively. Inserts of (**a**,**b**) are surface water drop profiles, inserts of (**c**,**d**,**e**,**f**) are corresponding Young’s moduli histograms. Each CA measurement or AFM image was conducted at least 3 times in different position of a film to ensure the reliability of data.

Above analysis indicated the good potential of lactose as a SA-specific receptor. Thus, the lactose units were grafted onto the PAM chain with the aid of benzene shiff base bridging units, and the grafting density was determined to be about 11% through the analysis of its ^1^H NMR spectra (see Supplementary Fig. S6). The acquired graft polymer PAM-*g*-lactose_0.11_ was further immobilized onto a flat silicon substrate through a “grafting to” approach, generating the PAM-*g*-lactose_0.11_ thin film. The as-prepared polymer film (average film thickness of 6 nm) displayed a relative even distribution of polymer chain with different chain-length suspended on surface, and surface topography with fluctuation in the range of 3~5 nm, as shown in the atomic force microscope (AFM) image in Fig. 3a (2 × 2 μm^2^) and Supplementary Fig. S7 (10 × 10 μm^2^). And the surface water contact angle (CA) was about 78°. This relative hydrophobic state of the polymer film could be attributed to the contracted polymeric network constructed by lactose and amide groups in PAM via hydrogen bonding interactions. Similar phenomena have been reported in our previous works^36^,^37^. After the PAM-*g*-lactose_0.11_ film being immersed in a Neu5Ac solution with a concentration of 0.02 mol · L^−1^ and a pH value of about 3.0 for 20 minutes, followed by removal of residual liquid and a subsequent drying process by nitrogen gas flow, obvious swelling in partial regions of the polymer film was observed in the AFM image (Fig. 3b), and the maximum surface fluctuation increased from 6 nm to 13 nm. Meanwhile, the polymer film became more hydrophilic, the CA decreased from 78° to about 54°. More interestingly, these remarkable changes in surface topography and CA of the polymer film could revert to the initial state upon the further treatment by neutral pure water. In sharp contrast, when the film was treated by a galactose or glucose solution under the same condition, no substantial changes in surface topography and CA were detected (see Supplementary Fig. S8), which revealed the high recognition selectivity of our polymer film towards Neu5Ac.

**Figure 4:**
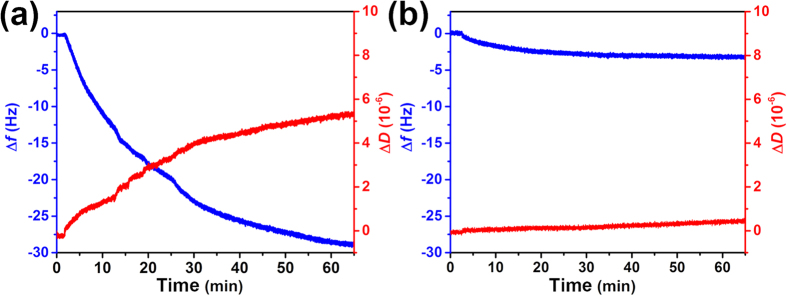
The adsorption investigation of the polymer film toward Neu5Ac and galactose. Time dependence of frequency change (Δ*f*) and dissipation change (Δ*D*) of QCM resonators grafted with the PAM-*g*-lactose_0.11_ polymer film upon adsorption of (**a**) Neu5Ac and (**b**) galactose.

The surface Young’s modulus images of the PAM-*g*-lactose_0.11_ film were also obtained by AFM in quantitative nano-mechanical property mapping (QNM) mode. Figure 3c and d show the surface Young’s modulus images of the polymer film before and after being treated by the Neu5Ac solution, in which clear colour change from green to red was observed. Meanwhile, statistical data indicated that the average Young’s modulus (mean ± s.d.) of the film surface decreased from the initial 270 ± 66 MPa to 110 ± 23 MPa, implying that the polymer film became much softer after interacting with Neu5Ac^38^. The surface Young’s modulus could also revert to its initial value upon the water treatment. By comparison, no obvious decreases in Young’s modulus for the film upon treatment by galactose (251 ± 51 MPa) and glucose (240 ± 46 MPa), respectively, were observed, as shown in Fig. 3e and f. To validate the role of lactose unit, wettability experiments based on the pure PAM polymer film was performed. Nevertheless, there was almost no substantial change in wettability of PAM film upon treatment by Neu5Ac (see Supplementary Fig. S9). This indicated that the specific CCI between the receptor lactose unit and Neu5Ac was the main driving force for the remarkable transitions in surface topography, wettability and stiffness of the polymer film. In addition, to further investigate the adsorption behaviors of this polymer film toward Neu5Ac and changes of the film itself, we performed an experiment based on Quartz Crystal Microbalance with Dissipation monitoring (QCM-D) by grafting the polymer PAM-*g*-lactose_0.11_ onto the resonator surface. And the results showed a strong Neu5Ac adsorption occurred, inducing a frequency change (Δ*f*) of the resonator of about 28 Hz in one hour, as shown in Fig. 4a. Meanwhile, the Neu5Ac adsorption also caused the obvious increase of energy dissipation (Δ*D*), which implied that the viscoelasticity and conformation of polymer film changed upon the adsorption of Neu5Ac^39^. By comparison, the pumping of galactose solution only caused a frequency change of the resonator of about 3 Hz and a negligible energy dissipation under the same condition (Fig. 4b). These results further confirmed the recognition selectivity of the polymer film towards Neu5Ac, and which resulted in the generation of the swollen polymer chains conformation and a more viscous surface property.

**Figure 5:**
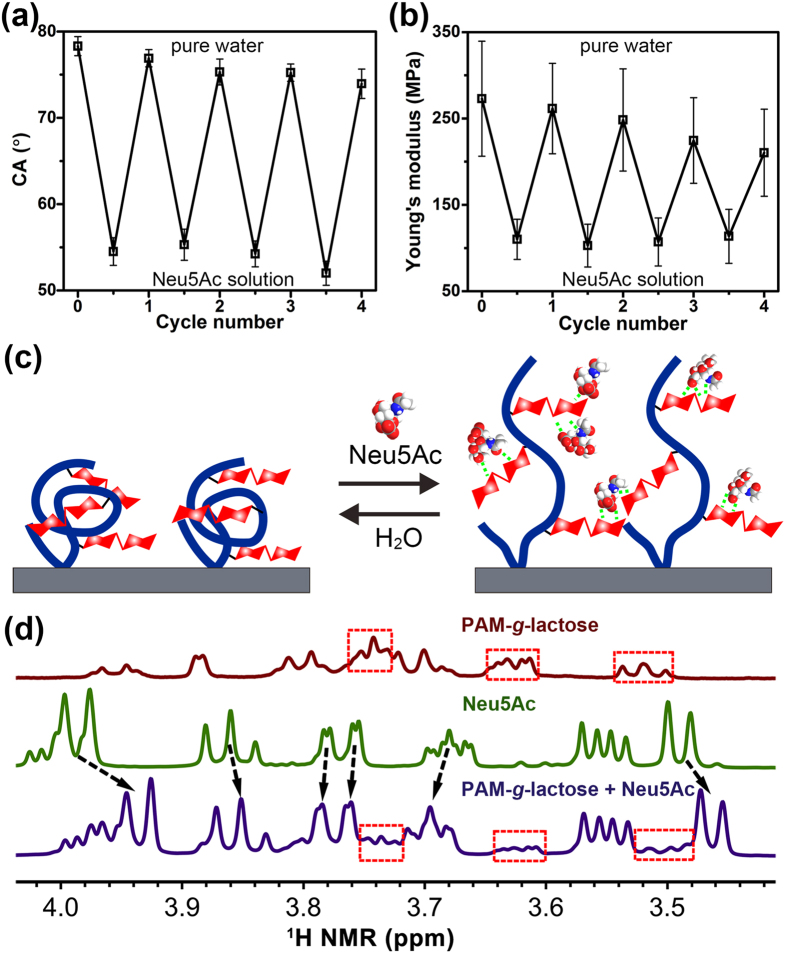
Reversible surface wettability and stiffness switching of the polymer film in response to the adsorption/desorption of Neu5Ac and possible conformation transformation mechanism of the polymer chains. (**a**) Cycle experiments illustrating reversible switching of contact angle (CA) and (**b**) AFM Young’s modulus of the PAM-*g*-lactose_0.11_ film upon alternative treatment by acidic Neu5Ac solution and neutral pure water. Each CA measurement or Young’s modulus image was conducted at least 3 times in different position of a film to ensure the reliability of data. (**c**) An ideal transformation model shows possible reversible contraction-swelling conformational change of the polymer chains induced by the adsorption/desorption of Neu5Ac. (**d**) Partial ^1^H NMR spectra of PAM-*g*-lactose_0.11_ (brown), Neu5Ac (green) and their mixture with a mass ratio of 1:1 (blue) in D_2_O at 20 °C. Clear shifts of some protons of the grafted lactose unit and Neu5Ac guests revealed the intensive hydrogen bond interactions between lactose and Neu5Ac.

Meanwhile, cycle measurements of CA and Young’s modulus revealed the good reversibility for macroscopic properties switching of the PAM-*g*-lactose_0.11_ film in response to the adsorption/desorption of Neu5Ac. As shown in Fig. 5a and b, the reversibility of CA and Young’s modulus of the polymer film was still maintained in the fourth cycle. Based on the above results and analyses, a possible step-by-step conformation transformation mechanism for polymer chains is proposed, as illustrated in Fig. 5c. Initially, the polymer chains exhibited a relative contracted form via intermolecular hydrogen bonds between lactose and adjacent lactose units or PAM amides. When immersed in Neu5Ac solution, exposed lactose sites in the terminal of polymer chain around surface preferred to bind and adsorb the Neu5Ac molecules via CCIs, this point further was confirmed by ^1^H NMR spectra of the complexation of PAM-*g*-lactose_0.11_ with Neu5Ac, where nearly all C-*H* protons of Neu5Ac displayed evident chemical shift change after interacting with the polymer, as shown in Fig. 5d. Then, such Neu5Ac adsorption broke the polymer chain’s endmost conformation and caused the exposure of more lactose units embedded in film, which further promoted the binding of newly exposed lactose units with more Neu5Ac molecules. As a result, the entire polymer chain was fully “open”, and the polymer film became a relative fluffy swelling state, accompanied by the increase in surface hydrophilicity and the decrease in surface stiffness. This presumption was further proven by the noticeable increase (about 6 nm) in the polymer film thickness (see Supplementary Fig. S10). Subsequent water treatment leaded to the desorption of the bound Neu5Ac due to the diffusion of Neu5Ac from the polymer network into surrounding water, thus the polymer chains could gradually revert to their initial states after collapse and reorganization of the polymer chain network. This biomolecule-modulated reversible switching in polymer conformation and surface properties may find wide applications in the fields of bio-sensing, bio-separation and tissue engineering^40^.

**Figure 6:**
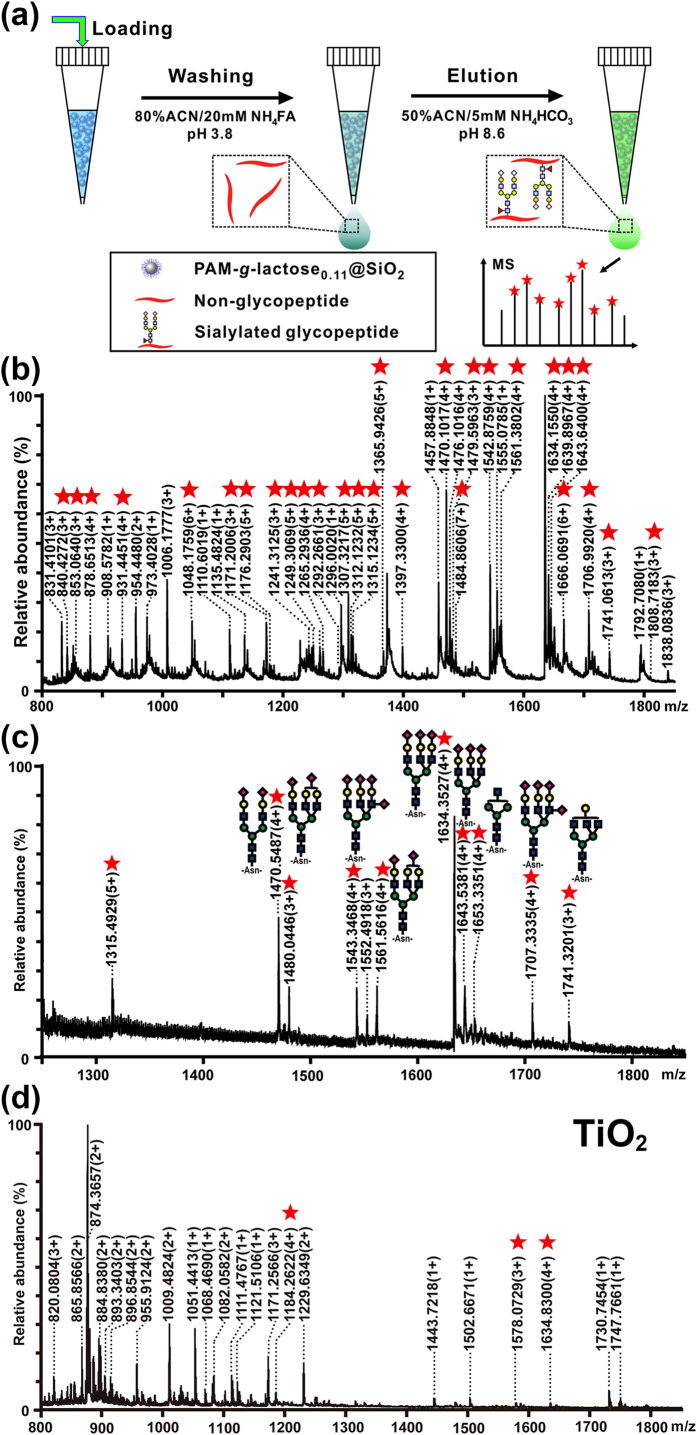
Glycopeptides enrichment performance of PAM-*g*-lactose_0.11_@SiO_2_. (**a**) pH-dependent enrichment procedure based on a micro-SPE mode. (**b**,**c**) Mass spectra of glycopeptides enriched with PAM-*g*-lactose_0.11_@SiO_2_ from tryptic digests of fetuin and BSA interference at molar ratios of 1:100 (**b**) and 1:300 (**c**). The non-glycopeptides are led with their m/z values, glycopeptides are marked with red stars or their glycan structures. ■: GlcNAc; green ●: mannose; yellow ●: galactose; ♦: Neu5Ac. Detailed peptide and glycan information is shown in Table S4 in Supplementary information. (**d**) Mass spectra of glycopeptides enriched with TiO_2_ from tryptic digests of fetuin and BSA at a molar ratio of 1:100.

Up to now, most of sialylated glycopeptides (SGs) enrichment methods are established generally based on chelation interaction between negatively charged SGs and TiO_2_^41^,^42^, saccharide-protein interaction between glycan moieties and lectins^43^,^44^, hydrophilic interaction between SGs and hydrophilic chromatographic stationary phases^45^,^46^, and covalent bond-formation (boronic acid-based materials or hydrazide chemistry reagents)^47^,^48^,^49^. However, each enrichment strategy displays its distinctive merits while usually also suffers from some inherent limitations, which has been well summarized in several excellent reviews^25^,^50^,^51^,^52^. Since the determination of great significance on SGs for immune disease diagnosis and cancer biomarkers discovery^53^,^54^,^55^, researchers have never ceased to pursue high-efficiency approaches for SGs enrichment. Herein, strong binding capacity of the PAM-*g*-lactose_0.11_ towards SA and reversible binding behaviour inspired us to apply this polymer material to enrich SGs. Firstly, the polymer PAM-*g*-lactose_0.11_ modified silica gels (denoted as PAM-*g*-lactose_0.11_@SiO_2_) was prepared (detailed preparation method is described in the experiment section). Then, tryptic digests of bovine fetuin (a standard glycoprotein) was employed as model sample to evaluate the enrichment selectivity of PAM-*g*-lactose_0.11_@SiO_2_, while different levels of digests from bovine serum albumin (BSA, a typical non-modified protein) was mixed with fetuin as interference in order to mimic complex biosamples. In addition, a facile stepwise enrichment method by controlling the pH of washing solution (pH = 3.8) and elution solution (pH = 8.6) buffers based on the micro-scale solid phase extraction (SPE) mode was established, as described in Fig. 6a^55^. We then evaluated the enrichment selectivity of PAM-*g*-lactose_0.11_@SiO_2_ for SGs in the micro-SPE mode, by using the tryptic digests of bovine fetuin mixed with different interference levels (e.g., 1:100 and 1:300 in molar ratios) of BSA interference. Before enrichment, the whole mass spectrum (MS) obtained from the tryptic digests of fetuin was occupied by non-glycopeptide signals and no glycopeptide signal could be detected. When the PAM-*g*-lactose_0.11_@SiO_2_ was evaluated, up to 28 glycopeptide signals were readily detected in MS after enrichment from the tryptic digests of fetuin mixed with 100-fold BSA interference, as shown in Fig. 6b. The good enrichment selectivity was still maintained even with 300-fold BSA interference (Fig. 6c). Furthermore, tandem MS/MS mass spectra of 7 glycopeptides had the indicator fragment ions of sialic acid, indicating that these signals were SGs (see Supplementary Fig. S12). However, in control experiments, PAM@SiO_2_ or Sepharose (a commercially available enrichment material) could not worked effectively when the interference level was higher than 10-fold BSA (see Supplementary Fig. S13) or 50-fold BSA (see Supplementary Fig. S14), respectively. Even the selective adsorbent for SGs, TiO_2_ demonstrated low specificity for SGs from the mixture of fetuin and BSA at the molar ratio of 1:100 (Fig. 5d). Therefore, these results clearly indicated the smart PAM-*g*-lactose_0.11_@SiO_2_ enabled highly selective enrichment towards SGs. To validate the feasibility of applying PAM-*g*-lactose_0.11_ @SiO_2_ for in-depth *N*-linked glycosylation profiling, our enrichment strategy was applied to a real biological sample for further evaluation. PAM-*g*-lactose_0.11_ was used to enrich glycopeptides from the tryptic digests of a HeLa S3 cell lysate. More than 140 glycopeptides with 119 unique glycosylation sites were successfully identified from 50 μg protein samples (the detailed glycosylation site information see Supplementary Table S5). This indicated the potential application of PAM-*g*-lactose_0.11_-based enrichment method in glycoproteome research in the near future.

**Figure 7:**
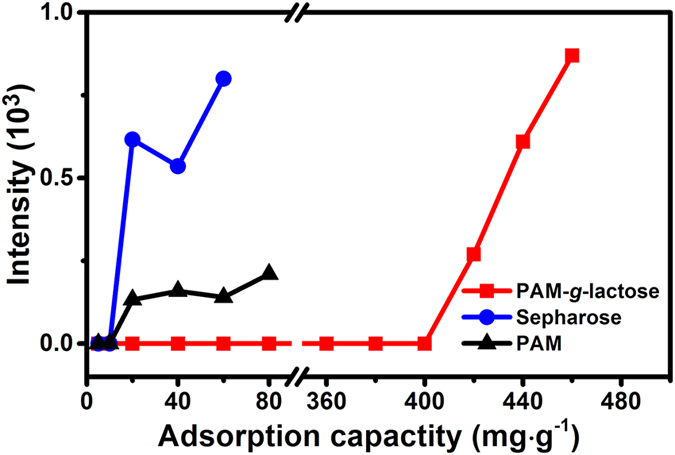
Determination of glycopeptides adsorption capacity of PAM-*g*-lactose_0.11_@SiO_2_ (red square), PAM@SiO_2_ (black triangle) and commercially available Sepharose materials (blue circle).

As another key parameter of enrichment material, adsorption capacity has a great significance for large-scale and high-throughput glycoproteomic studies^56^. Thus we further evaluated the adsorption capacity of PAM-*g*-lactose_0.11_@SiO_2_ towards glycopeptides. As illustrated in Fig. 7, our material showed a high adsorption capacity of about 400 mg · g^−1^ towards glycopeptide in the tryptic digests of fetuin, which is much higher than that of Sepharose (about 10 mg · g^−1^) and the largest adsorption capacity (100 mg · g^−1^) reported in the literatures^57^. In a control experiment, PAM@SiO_2_ only showed a rather low adsorption capacity of about 10 mg · g^−1^. Therefore, this high adsorption capacity of our polymeric material could be reasonably attributed to the abundant lactose-based binding sites for SGs, as well as the relaxed polymer chains facilitating the binding of lactose with more SGs. Since the relative hydrophobic state of the polymer film with a water contact angle of 78°, we presume that hydrophilic interaction between lactose units and SGs might not be the main driving force.

## Conclusion

In summary, we developed a novel smart polymer PAM-*g*-lactose_0.11_ thin film, which exhibited high and specific binding capacity towards Neu5Ac promoted by the distinctive carbohydrate-carbohydrate interactions (CCIs) between grafted lactose units and target SA molecules, as well as the binding-triggered remarkable and reversible switching in surface topography, wettability and stiffness on the polymer film. With these features, the prepared PAM-*g*-lactose_0.11_@SiO_2_ material further achieved the highly selective enrichment toward SGs with strong anti-interference ratio of 1:300 and high adsorption capacity of 400 mg · g^−1^. Therefore, this innovative utilization of the CCI as a unique binding force for SA recognition, reversible transition of material macroscopic properties and satisfactory enrichment application give a glimpse of the significance of this distinctive biomolecule interaction, which might will spark people’s interests on CCI in various bio-related applications. For example, due to over-expressed SA-containing glycans in tumor cells surface and acidic intratumor microenvironment, this specific SA recognition capacity of our polymer material may provide a new methods to develop tumor-targeting drug delivery system^21^. Moreover, it is also conceivable that this smart polymer shows potential for early diagnosis and monitor of cancers by reversibly capture and release circulating tumor cells^22^. In addition, on the basis of CCI mechanism, the design strategy by integrating receptor into flexible polymer chains endowed the material with excellent switchable properties in response to biomolecules, offering an encouraging approach for the design of smart biointerface materials. In addition, the excellent enrichment selectivity and high adsorption capacity demonstrate the great potential of our smart polymer in glycopeptides enrichment, which provide some enlightenment for the development of high-performance glycopeptide enrichment materials^58^,^59^.

## Methods

### Materials

Polyacrylamide (PAM, weight-average molecular weight: 10000, Sigma-Aldrich), D-(+)-lactose (lactose, 98%, TCI), bovine fetuin (Sigma-Aldrich), bovine serum albumin (BSA, Sigma-Aldrich) were used as received. Silicon wafer (P type, 1-0-0, MCL electronic materials, Ltd., Luoyang), silica gels (average particle size: 5 μm, inner pore size: 300 Å, Fuji Silysia Chemical, Ltd.) were cleaned before use according to standard procedures. Various monosaccharides including D-(+)-glucose (glucose), D-(+)-galactose (galactose), *N*-acetyl-D-glucosamine (GlcNAc), *N*-acetyl-D-galactosamine (GalNAc), D-(+)-mannose (mannose), L-(−)-fucose (fucose), *N*-acetyl-neuraminic acid (Neu5Ac), and gluconic acid, ascorbic acid, tartaric acid were used as received from TCI Corp. Double distilled water (18.2 MΩ · cm, MilliQ system) was used, and other general solvents and chemicals were used as received.

### Experimental investigations of the lactose-based receptor for SA

Firstly, fluorescein-labelled lactose molecule was chosen as host to perform the fluorescent titration experiments towards different monosaccharides (see Supplementary information for more experimental details). The lactose was prepared as stock solution in Tris-buffer solution (10 mM, pH 7.4) and formate-buffer solution (10 mM, pH 3.8) for 5.0 × 10^−6^ mol · L^−1^. Guest monosaccharides were prepared to 1.75 × 10^−3^ and 1.75 × 10^−2^ mol · L^−1^ of stock solutions in H_2_O. The work solutions were prepared by adding different volumes of guest solution to a series of test tubes, and then same amount of stock solution of host FITC-labelled lactose was added into each test tube, followed by dilution to 3 mL by corresponding buffer solution. After being shaken for 1 minute, the work solutions were measured immediately at 20 °C using the spectrophotometer. Each measurement was repeated 3 times to ensure the reliability of data.

### Synthesis of the polymer PAM-*g*-lactose_0.11_

PAM (2.5 g), Na_2_CO_3_ (0.5 g) and formyl-phenyl β-D-lactoside (1 g, for the preparation process see Scheme S2 in Supplementary information) were dissolved in a mixture solution of 10 mL H_2_O and 15 mL MeOH. After stirring for 48 hours at 60 °C, the crude product was purified by dialysis in H_2_O/MeOH mixture for 3 days using dialysis membrane (molecule weight cut-off: 10000). The target product PAM-*g*-lactose_0.11_ was obtained as white powder after freeze-drying. The grafting density of lactose units is about 11% according to its ^1^H NMR (see Supplementary Fig. S6).

### Preparation of PAM-*g*-lactose_0.11_ thin film on the silicon substrates

Silicon wafers (10 × 10 mm) were cleaned by immersing in a fresh mixture of H_2_SO_4_ (98%) and H_2_O_2_ (30%) (7:3, v/v) for 30 minutes at 100 °C. Then, the cleaned wafers were immersed in a NaOH solution (0.1 mol · L^−1^) for 8 minutes and subsequently in HNO_3_ (0.1 mol · L^−1^) for another 15 minutes. After being rinsed with an excess of double distilled water and dried under a flow of nitrogen gas, silicon wafers were allowed to react with 3-triethoxysilylpropyl isothiocyanate (500 μL) in 10 mL dry toluene for 6 hours at 60 °C, to obtain the chemically bonded isothiocyanate-activated (NCS active) groups on the silicon substrates. These substrates were rinsed again with anhydrous toluene and dichloromethane for three times to remove the remaining reactants. After being dried under a flow of nitrogen gas, the substrates were immersed in 10 mL double distilled water containing 0.10 g PAM-*g*-lactose_0.11_ for 24 hours at 40 °C, allowing a coupling reaction between the amide residues in PAM and the active NCS sites on the silicon substrates to occur. After that, the substrates were rinsed with double distilled water for three times to remove the unbound PAM-*g*-lactose_0.11_, and dried under a flow of nitrogen gas. By utilizing the same method, pure PAM thin film was also grafted onto the silicon substrates. PAM-*g*-lactose_0.11_@SiO_2_ and pure PAM@SiO_2_ was obtained by using the similar method (see Supplementary information for more details).

### Contact angle measurements

The silicon substrate with PAM-*g*-lactose_0.11_ thin film was placed on the sample stage of DataPhysics OCA35 goniometer, and a water droplet of 3 μL was carefully deposited on the film surface by a precise electric dosing syringe, then surface water contact angle (CA) of PAM-*g*-lactose_0.11_ thin film was recorded using the sessile drop method at ambient atmosphere and a constant temperature of 25 °C. Each measurement was repeated 3 times to ensure the reliability of data.

### AFM measurements

The surface topography and stiffness of PAM-*g*-lactose_0.11_ thin film was investigated by AFM in QNM mode. The system was calibrated by using the absolute method recommended by Bruker’s user manual before each experiment. On scan parameters, ScanAsyst Auto Control was set to ON, scan rate was set at 1 Hz. Firstly, the as-prepared PAM-*g*-lactose_0.11_ thin film sample was measured and obtained an original result. Then, the film was immersed in monosaccharide solutions with an identical concentration of 0.02 mol · L^−1^ for 20 minutes. After that the surface was dried under a flow of nitrogen gas to remove any remaining excess liquid, then AFM measurement was performed, and AFM images of surface topography and stiffness were obtained. Each measurement was conducted at least 3 times in different position of a film.

### Enrichment of glycopeptides

Glycopeptide enrichment was performed in a micro-scale SPE mode. Firstly, an Eppendorf GELoader tip was packed with 1 mg PAM-*g*-lactose_0.11_@SiO_2_ material after being slurried with acetonitrile (ACN) to obtain the SPE micro-column. Then the tip was conditioned and equilibrated with 20 μL 50%ACN/20 mM ammonium formate (NH_4_FA) (pH 3.8) and 20 μL 80%ACN/20 mM NH_4_FA (pH 3.8), respectively. When tryptic digests dissolved in 80%ACN/20 mM NH_4_FA was loaded, the micro-column was washed twice with 20 μL 75% ACN/20 mM NH_4_FA (pH 3.8) and 20 μL 75%ACN/0.1%formic acid (FA), respectively. Subsequently, the captured glycopeptides were eluted with 30 μL 50%ACN/5 mM NH_4_HCO_3_ (pH 8.6). Similar enrichment procedure was also employed for reference material PAM@SiO_2_. As for commercial material control experiments, TiO_2_^55^ and Sepharose^60^ were used to enrich SGs, performed according to the reported method with minor modification.

### Adsorption capacity measurement

Fetuin tryptic digest was dissolved in 80% ACN/20 mM NH_4_FA to final concentration of 0.1 mg·mL^−1^. And a series of 20 μL tryptic digests were successively loaded into the SPE micro-column prepacked with 1 mg materials. Each elution fraction from the micro-column was collected and analyzed with MS. Before the point of the adsorption capacity, no glycopeptide signal could be detected in elution fraction. When the amount of glycopeptides sample loaded into the SPE micro-column is beyond the adsorption capacity, the target signal could be detected. The adsorption capacity was determined before the point that the target glycopeptide signal was detected. With this method, adsorption capacity of Sepharose and PAM@SiO_2_ were also measured.

## Additional Information

**How to cite this article**: Xiong, Y. *et al*. Sialic Acid-Responsive Polymeric Interface Material: From Molecular Recognition to Macroscopic Property Switching. *Sci. Rep.* **7**, 40913; doi: 10.1038/srep40913 (2017).

**Publisher's note:** Springer Nature remains neutral with regard to jurisdictional claims in published maps and institutional affiliations.

## Supplementary Material

Supplementary Information

Supplementary Dataset 1

Supplementary Dataset 2

## Figures and Tables

**Figure 1:**
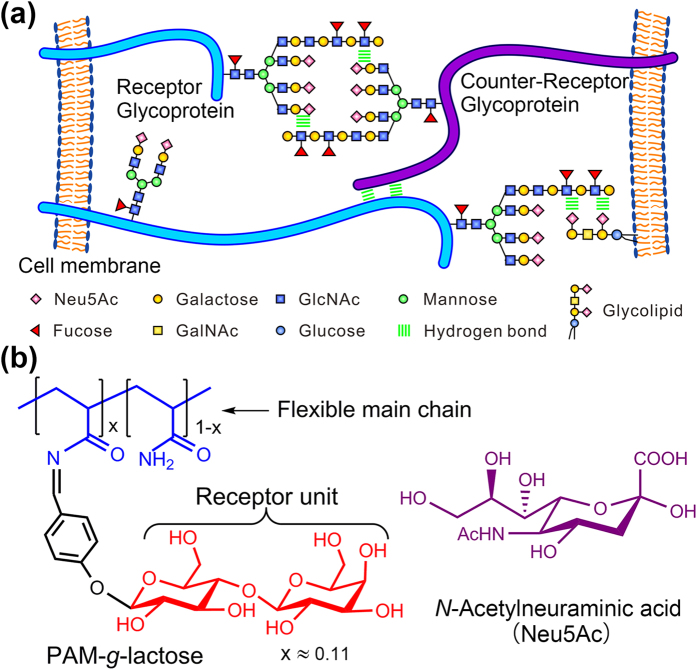
Sialic acid-responsive polymer design idea inspired from carbohydrate-carbohydrate interaction. (**a**) Schematic illustration of carbohydrate-carbohydrate interactions occurred among glycoconjugates (i.e., glycoprotein, glycolipid, and glycans) located on cell membrane surface^26^. (**b**) Chemical structures of grafted polymer PAM-*g*-lactose_0.11_, and Neu5Ac (a typical sialic acid).
