# Supplementary material for: Sialic Acid-Responsive Polymeric Interface Material: From Molecular Recognition to Macroscopic Property Switching
Source: Sci Rep. 2017 Jan 13;7:40913. doi: 10.1038/srep40913 (PMC5234036; doi:10.1038/srep40913)
Supplement: Supplementary Information [file srep40913-s1.pdf]

Supplementary Information

Sialic Acid-Responsive Polymeric Interface Materials: From Molecular Recognition to Macroscopic Property Switching

Yuting Xiong[1], Ge Jiang[2], Minmin Li[1], Guangyan Qing[1,*], Xiuling Li[2,4,*], Xinmiao Liang[2,4] and Taolei Sun[1,3]

[1] State Key Laboratory of Advanced Technology for Materials Synthesis and Processing, Wuhan University of Technology, 122 Luoshi Road, Wuhan 430070, P. R. China. E-mail: qing@whut.edu.cn

[2] Key Laboratory of Separation Science for Analytical Chemistry, Dalian Institute of Chemical Physics, Chinese Academy of Sciences, 457 Zhongshan Road, Dalian 116023, P. R. China. E-mail: lixiuling@dicp.ac.cn

[3] School of Chemistry, Chemical Engineering and Life Science, Wuhan University of Technology, 122 Luoshi Road, Wuhan 430070, P. R. China

[4] Co-innovation Center of Neuroregeneration, Nantong University, Nantong, 226019, P. R. China.

Part S1 Supporting experimental details

Additional materials and reagents: Ammonium bicarbonate (NH_4HCO_3), urea, DL-dithothreitol (DTT), ethylene diaminetetraacetic acid (EDTA), iodoacetamide (IAA), ammonium carbonate, trypsin, fluorescein isothiocyanate and Tris were purchased from Sigma-Aldrich Corp. (St. Louis, MO, USA). Dimethyl formamide (DMF), dimethylsulfoxide (DMSO), methanol, ethanol, chloroform, pyridine, dichloromethane and triethylamine (Et_3N) were purchased from Alfa Aesar Corp. (Tianjin, China). Tetrabutylammonium iodide and sodium methoxide with high purities (>99%) were purchased from TCI (Tokyo, Japan). 3-Triethoxysilylpropyl isothiocyanate (97%) was purchased from Meryer Chemical Technology (Shanghai, China). Double distilled water (18.2 MΩ·cm, MilliQ system) was used. GELoader tips were obtained from Eppendorf (Madison, WI, Germany).

Instruments: Fluorescence spectra were recorded on a PerkinElmer LS55 Fluorescence Spectrophotometer. NMR spectra were recorded on Varian Mercury VX 300-MHz spectrometer and Bruck AVANCE III 500-MHz spectrometer. X-ray photoelectron spectroscopy (XPS) was obtained with a VG Multilab 2000. FI-IR was performed with a Bruker Vertex 80V FT-IR spectrometer. Scanning Electron Microscopy (SEM) spectra were recorded on a Hitachi S-4800 SEM. Mass spectra were acquired with a nano Electrospray Ionization-Quadrupole Time-of-Flight mass spectrometer (ESI Q-TOF MS) and LTQ Orbitrap Velos coupled with Accela 600 HPLC system. Atomic force microscopy (AFM) investigations were conducted via Bruker Multimode 8 AFM in the ScanAsyst mode and quantitative nanomechanical property mapping (PeakForce QNM) mode. Water contact angle (CA) measurements were conducted using the sessile drop method on a DataPhysics OCA35 goniometer with software SCA20. Dynamic adsorption experiments were performed on a quartz crystal microbalance with dissipation monitoring (QCM-D) (Q-Sense E4 System, Biolin Scientific Corp. Sweden)

Synthesis of fluorescein-labelled lactose

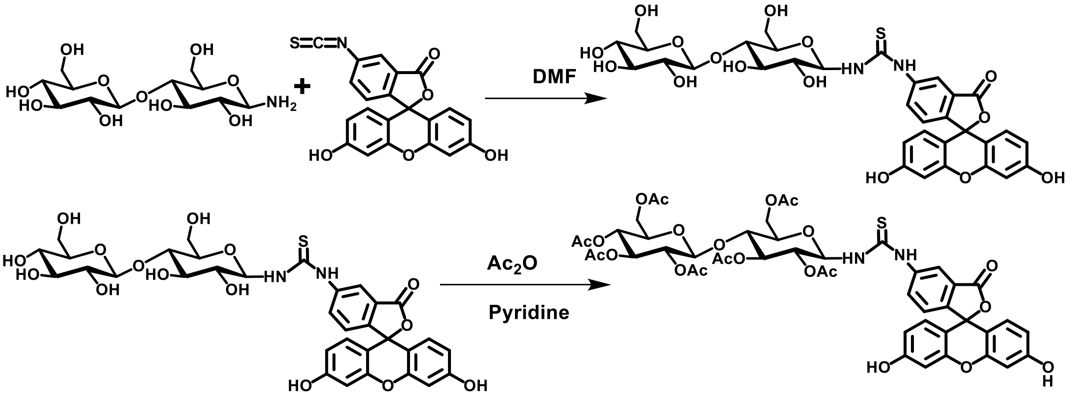

Scheme S1. Synthesis of fluorescein-labelled lactose

Automated microwave tube (30 mL) was charged with 2.05 g D-lactose (denoted as lactose) (6.0 mmol), 5.0 g ammonium carbonate and 15 mL anhydrous DMSO. The tube was sealed and placed in an automated microwave synthesizer (Biotage Initiator 8 EX), the reaction was proceeded at 40 ℃ in 10 watts power for 4 hours. Then the reaction mixture was freeze-dried to remove the excess of ammonia and DMSO to obtain β-glycosyl amine (1-Amino-lactose).[1] Then 0.34 g β-glycosyl amine (1-Amino-lactose 1.0 mmol), 0.39 g fluorescein isothiocyanate (FITC, 1.0 mmol), and 5 mL anhydrous DMF were added to a 25 mL round-bottom flask. The reaction mixture was stirred at room temperature overnight. After that the solvent was removed under reduced pressure. The crude product was purified through a Shimadzu UFLC 20A purity system using a C18 reversed-phase analytic chromatographic column (10 mm×250 mm, Boston Analytics, Corp. China) to afford fluorescein-labelled lactose as yellow power 0.527 g, yield: 72%. Hydrogen nuclear magnetic resonance (^{1}H NMR, 300 MHz, CD_3OD) δ (ppm): 3.45-3.51 (m, 3H, Sugar ring-*CH*), 3.54-3.64 (m, 7H, Sugar ring-*CH*), 3.69-3.73 (m, 2H, Sugar ring-*CH*), 3.79-3.84 (m, 2H, Sugar ring-*CH*, 1H, OH), 3.88-3.90 (m, 2H, *OH*), 4.33-4.39 (m, 2H, OH), 4.58-4.60 (m, 2H, *OH*), 6.52, 6.55 (d, d, J_1=J_2=2.1 Hz, 3H, Ar-*H*), 6.64-6.68 (m, 5H, Ar-*H*), 7.15 (d, *J*=8.4 Hz, 1H, Ar-*H*), 7.46 (s, 1H, Ar-*OH*), 7.80-7.84 (m, 1H, Ar-*OH*), 7.91 (s, 1H, CSN*H*), 8.20 (s, 1H, CSN*H*). Infrared spectrum (IR): 3213, 1668, 1581, 1532, 1488, 1456, 1366, 1301, 1272, 1235, 1201, 1170, 1116, 1076, 1013, 950, 849, 799, 761, 722, 660, 642, 596 cm^{-1}. MADLI MS: m/z calcd for $C_{33}H_{34}N_2O_{15}S$: 730.17; found: 732.20 $[M+2H]^+$. Elemental analysis calcd. (%) for $C_{33}H_{34}N_2O_{15}S$: C, 54.24; H, 4.69; N, 3.83; S, 4.39. Found: C, 54.15; H, 4.79; N, 3.87; S, 4.51.

The above fluorescein-labelled lactose (0.366 g, 1 mmol) was dissolved in 5 mL dry pyridine, then acetic anhydride (0.714 g, 7 mmol) was added to the above solution and kept stirring for 12 hours at ambient temperature. Subsequently, the reaction solution was poured into an ice water (50 mL), the precipitate was washed with ice water and petroleum ether for three times. After that the crude product was purified through Shimadzu UFLC 20A purity system using a C18 reversed-phase chromatographic column, to afford fluorescein-labelled acetylated lactose as yellow power 0.369 g, yield: 36%. ^{1}H NMR (300 MHz, $CDCl_3$) δ (ppm): 1.59 (s, 3H, COC*H*$_3$), 2.60, 2.65 (d, d, J_1=J_2=4.8 Hz, 2H, Sugar ring-C*H*), 2.79 (d, *J*=3.9 Hz, 1H, Sugar ring-C*H*), 2.87 (s, 9H, COC*H*$_3$), 3.05 (t, J_1=J_2=5.7 Hz, 3H, Sugar ring-C*H*), 3.31, 3.74 (s, s, 9H, COC*H*$_3$), 4.12-4.15 (m, 2H, Sugar ring-C*H*), 4.70-4.76 (m, 2H, Sugar ring-C*H*), 5.94 (d, *J*=7.2 Hz, 2H, Sugar ring-C*H*), 6.11 (d, *J*=8.4 Hz, 2H, Sugar ring-C*H*), 7.02-7.04 (m, 3H, Ar-*H*), 7.16 (d, *J*=7.8 Hz, 2H, Ar-*H*), 7.47-7.59 (m, 3H, Ar-*H*), 8.21, 8.24 (d, d, J_1=7.5 Hz, J_2=8.7 Hz, 1H, Ar-*H*, 2H, Ar-O*H*), 8.51 (d, *J*=8.4 Hz, 2H, CSN*H*). IR: 2937, 1747, 1664, 1608, 1493, 1420, 1367, 1195, 1151, 1109, 1074, 1034, 1013, 891, 692, 675, 600 cm^{-1}. MADLI MS: m/z calcd for $C_{47}H_{48}N_2O_{22}S$: 1024.24; found: 1026.24 $[M+2H]^+$. Elemental analysis calcd. (%) for $C_{47}H_{48}N_2O_{22}S$: C, 55.08; H, 4.72; N, 2.73; S, 3.13. Found: C, 55.00; H, 4.79; N, 2.78; S, 3.20.

Synthesis of the polymer PAM-*g*-lactose

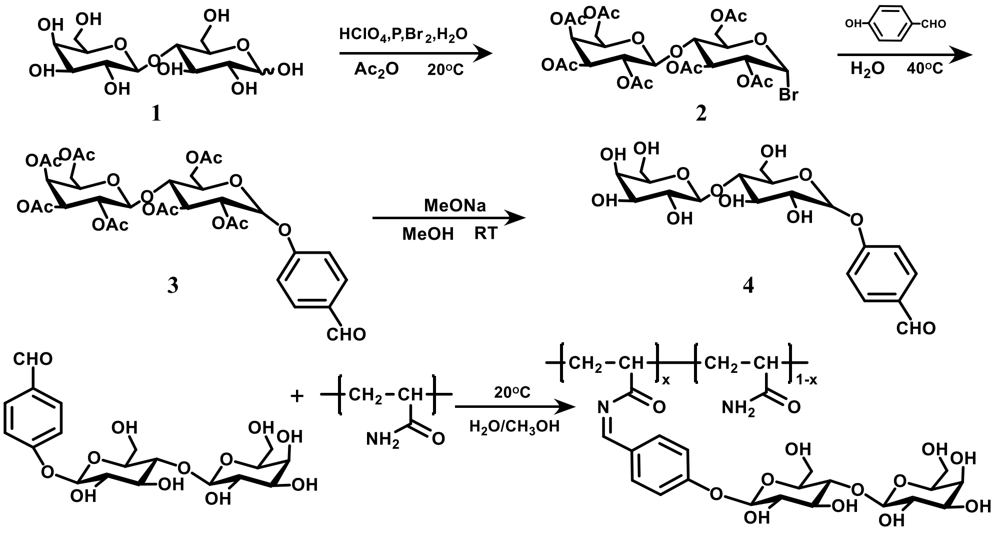

Scheme S2. Synthetic procedure of PAM-*g*-lactose.

Lactose **1** (34 g, 0.1 mol) was added into a 500 mL three necked RB flask containing Ac_2O (160 mL) and $HClO_4$ (1 mL) at 20 ℃. The reaction mixture was stirred for 2.5 hours while maintaining reaction temperature below 30 ℃. After that, the reaction mixture was placed in an ice bath and red phosphorus (12.0 g), Br_2 (20 mL) and distilled H_2O (14 mL) were added in sequence while maintaining reaction temperature below 20 ℃. The ice bath was removed and the reaction was continued for another 4 hours at room temperature. The mixture was diluted with chloroform (120 mL), poured into ice water, neutralized with saturated aqueous Na_2CO_3. The combined organic layer was dried over $CaCl_2$ and concentrated under vacuum to afford 40 g crude yellow oil product **2** (75%) which could be used without further purification.

To a 500 mL three necked RB flask containing a mixture of $CHCl_3$ (75 mL), distilled H_2O (75 mL), and tetrabutylammonium iodide (7.38 g, 0.02 mol) at 40 ℃, 100 mL $CHCl_3$ solution of **2** (69.9 g, 0.1 mol) and 100 mL aqueous solution of 4-hydroxybenzaldehyde (12.2g, 0.1 mol) were added dropwise at the same rate over 1.5 h. The mixture was stirred vigorously at 60 ℃ for 6 h. The organic layer was separated, washed with 5% aqueous NaOH (3 × 50 mL), dried ($MgSO_4$), and the solvent was evaporated under reduced pressure. The crude product was purified by column chromatography (petroleum ether–ethyl acetate, 5:2, v/v) to give 20.5 g of **3** as yellow oil (29% yield).[2]

To a solution of compound **3** (7.40 g, 10 mmol) in 20 mL of methanol was added 20 mL of methanol solution of 0.2 $mol \cdot L^{-1}$ sodium methoxide. The mixture was stirred for 3 h at room temperature. The methanol was removed under reduced pressure, and the residue was purified by chromatography (MeOH/$CHCl_3$, 1/9, v/v) to give pure 4-formylphenyl β-D-lactoside **4**, white powder, yield 89%.[3]

A 50 mL round-bottom flask was added a certain amount of 4-formylphenyl β-D-lactoside 1.0 g, polyacrylamide (PAM) 2.5 g, sodium carbonate 0.5 g, 10 mL water and 15 mL methanol.[4]

After stirring for 48 hours at 60 ℃, the crude product was obtained, and purified by dialysis in water and methanol for 3 days, using a piece of dialysis membrane (2 cm×10 cm, molecule weight cut-off: 10000). White powder of PAM-*g*-lactose was obtained after freeze-drying. ^{1}H NMR spectra was shown in Fig. S6. ^{1}H NMR (500 MHz, D_2O) : δ (ppm): 1.62 (m, 18H, C-CH_2), 2.19 (d, 9H, C-C*H*), 3.36-3.96 (m, 11H, C*H*-OH and CH_2-OH), 4.41 (t, *J*=3.0 Hz, 1H, C*H*-OH), 5.11(d, J=7.9 Hz, 1H, O-C*H*-O), 5.24 (d, *J*=7.8 Hz, 1H, O-C*H*-O), 7.21 (d, *J*=8.9 Hz, 2H, Ph-*H*), 7.89 (d, *J*=8.8 Hz, 2H, Ph-*H*), 9.76 (s, 1H, C*H*=N). IR (cm^{-1}): 3335, 3186, 2931, 2885, 1654, 1606, 1562, 1449, 1403, 1346, 1320, 1184, 1118, 1076, 1043. Elemental analysis for PAM-*g*-lactose found: C, 54.24; H, 7.32; N, 12.10.

Synthesis of PAM-*g*-lactose@SiO_2 enrichment material

(a) Silica gels (5.0 g, average particle size: 5 μm and average pore diameter: 300 Å) were suspended in 25 mL of hydrochloric acid (HCl, 0.1 mol·L^{-1}) for 48 hours at ambient temperature to generate sufficient hydroxyl groups on the silica surface. The hydroxyl activated silica gels were separated by centrifugation at 7000 rpm for 5 minutes. Then, the silica gels were washed three times with ultrapure water and then ethanol by repetitive dispersion/precipitation cycles, and then the silica gels were dried under vacuum.

(b) 3-triethoxysilylpropyl isothiocyanate (3.0 mL) was dissolved in anhydrous toluene (40 mL), and the aforementioned silica gels (5.0 g) were added. The mixture was stirred and refluxed for 6 hours. The product was separated by centrifugation at 7000 rpm for 5 minutes. Then, the isothiocyanate-modified silica gels (denoted as NCS@SiO_2) were washed three times with toluene and then ethanol by repetitive dispersion/precipitation cycles to remove the unreacted materials, and then the silica gels were dried under vacuum.

(c) 0.20 g PAM-*g*-lactose was allowed to react with 0.50 g 3-triethoxysilylpropyl isothiocyanate-modified SiO_2 microspheres (denoted as NCS@SiO_2)[5] in distilled water for 2 days, via a coupling reaction between the amide residues in PAM and the active NCS sites on the silica gels. Pure PAM-*g*-lactose@SiO_2 was obtained through alternate dialysis (2 cm×10 cm) with methanol and distilled water for three times.

Reference material PAM@SiO_2 was also obtained by using the same method.

Fluorescent titration experiment details

To investigate the binding properties (K_a) of lactose towards various saccharides, we used the fluorescein isothiocyanate (FITC) to label the lactose molecule to perform the fluorescent titration experiment, which is a typical and rapid method for measuring affinity constant between host and guest molecules in supramolecular chemistry.[6] Here, fluorescent titration experiments between fluorescein-labelled lactose and different saccharides, including galactose, fucose, glucose, mannose, GalNAc, GlcNAc, and Neu5Ac, were conducted in buffer solutions with pH 7.4 and 3.8, respectively. The host fluorescein-labelled lactose was prepared as stock

solution in Tris-buffer solution (10 mM, pH 7.4) and formate-buffer solution (10 mM, pH 3.8) for 5.0×10^{-6} mol L^{-1}, respectively. Guest monosaccharides were prepared to 1.75×10^{-3} and 1.75×10^{-2} mol L^{-1} of stock solutions in H_2O. The work solutions were prepared by adding different volumes of guest solutions to a series of test tubes, and then same amount of stock solution of host fluorescein-labelled lactose was added into each test tube, followed by dilution to 3.00 mL by Tris-buffer solution or formate-buffer solution. After being shaken for 1 minute, the work solutions were measured immediately at 20 °C using a spectrometry. Association constant (K_a) were obtained according to intensity changes at the maximum emission peak. Detailed K_a values are listed in Table S1.

Similar method was adopted to investigate the binding capacities of fluorescein-labelled maltose and fluorescein-labelled cellobiose to Neu5Ac and glucose in formate-buffer solution (10 mM, pH 3.8).

Similar method was adopted to investigate the binding capacities of fluorescein-labelled acetylated lactose to diverse monosaccharides in Tris-buffer solution, respectively. The detailed K_a values are shown in Table S2.

Moreover, the binding capacities of the fluorescein-labelled lactose towards three other typical acidic analogues (gluconic acid, ascorbic acid and tartaric acid) were also investigated in formate-buffer solution (10 mM, pH 3.8).

AFM measurement details

The general characterizations (film morphology and thickness) of the polymer film were performed using AFM in the ScanAsyst mode with a Nanoscope V controller and the software Nanoscope v8.12. The resulting images were processed using Nanoscope Analysis v1.40.

The surface stiffness of copolymer film was investigated by AFM in PeakForce QNM mode at ambient atmosphere and a constant temperature of 25 ℃. AFM with PeakForce QNM mode allows quantitative nanomechanical mapping of material surface properties, including Young's modulus, while simultaneously imaging the topography of sample at a high resolution.

In the PeakForce QNM mode, the reduced modulus E* is obtained by fitting the retraction curve using the Derjaguin-Muller-Toporov (DMT) model:[7]

$$F = \frac{4}{3}E^{*}\sqrt{R(d-d_0)^3} + F_{\mathrm{Adh}} \qquad \text{eq. 1}$$

Where F is the force on the tip, F_{Adh} is the adhesion force, R is the tip end radius, d-d_0 is the distance between tip and sample. As a soft polymer brush film, we used 0.4 as the sample's Poisson ratio as recommended by Bruker's users guide for a sample with stiffness lower than 1 GPa. Since the sample's Poisson ratio is known, the Young's modulus of the sample (Es) can been calculated by the following equation:

$$E^{*} = \left[\frac{1-\nu_{\mathrm{tip}}{}^2}{E_{\mathrm{tip}}} + \frac{1-\nu_{\mathrm{s}}{}^2}{E_{\mathrm{s}}}\right]^{-1} \qquad \text{eq. 2}$$

Where v_{tip} and E_{tip} are the Poisson's ratio and Young's modulus of the tip, v_s and E_s are the Poisson's ratio and Young's modulus of the sample. We assumed that the tip modulus E_{tip} is infinite, and calculate the sample Young's modulus using the sample Poisson's ratio.

In this work, a standard SNL-10 $A^{\#}$ probe was used and calibrated by using the absolute method (recommended by Bruker's PeakForce QNM users guide, 004-1036-000) before each experiment. The deflection sensitivity (61.50 nm/V) was measured on a clean silicon wafer surface, the spring constant was 0.1937 N/m by using thermal tuning method, the tip end radius (3.8 nm) was determined by scanning a TipCheck sample. On scan parameters, ScanAsyst Auto Control was set to ON, Scan rate was set at 1 Hz. Each scan was performed in an area of 2×2 μm^2 with a resolution of 512×512 data points. Firstly, the initial result of as-prepared PAM-*g*-lactose film was measured. Then, the polymer film was immersed in saccharide solutions (Neu5Ac, glucose, galactose) with a concentration of 0.02 mol L^{-1} for 20 minutes. After that the surface was dried under a flow of nitrogen gas to remove any remaining excess liquid, and then the mechanical properties of the polymer film were recorded by AFM in PeakForce QNM mode. The obtained images were processed using NanoScope Analysis v1.40.

For the cycling experiment, a cycle includes the alternative measurement after the polymer film was immersed in sugar solution of 2.0×10^{-2} mol·L^{-1} for 20 minutes and pure water for 20 minutes, respectively.

^{1}H NMR experiments

In order to investigate the binding details between lactose and Neu5Ac, ^{1}H NMR experiments were conducted in DMSO-d_6 and D_2O. Lactose was allowed to mix with Neu5Ac at a molar ratio of 1:1, concentrations: 2 mmol in DMSO-d_6 and D_2O, respectively. After equilibration for 4 hours, the chemical shifts were recorded by NMR spectrometer, as shown in Fig. S1 and Fig. S2. Similar ^{1}H NMR experiments were also conducted in D_2O to investigate the complexation of lactose with galactose and glucose at a molar ratio of 1:1, as shown in Fig. S4 and Fig. S5, respectively.

QCM adsorption experiment

PAM-*g*-lactose modified QCM-D resonator was first prepared. An Au-coated QCM resonator was firstly cleaned by a fresh mixture solution of distilled water, ammonium hydroxide and H_2O_2 (v/v/v, 5/1/1) for 10 minutes at 70 ℃. After being rinsed by double distilled water sufficiently and dried under a flow of nitrogen gas, the resonator was immersed in 10 mL water containing 2-mercaptoacetic acid 0.092 g (1 mmol) for 12 hours. After being rinsed by double distilled water sufficiently and dried under a flow of nitrogen gas, the resonator modified with 2-mercaptoacetic acid was immersed in 10 mL water containing 0.02 g PAM-*g*-lactose and *N*-(3-Dimethylaminopropyl)-*N*'-ethyl-carbodiimide (EDC, 0.04 g, 0.21 mmol) for 12 h. After being rinsed by double distilled water sufficiently and dried under a flow of nitrogen gas, PAM-*g*-lactose modified QCM-D resonator was obtained.

The QCM adsorption experiment was carried out on a Q-Sense E4 system (Sweden). The Au-coated QCM resonators with intrinsic frequency (F_0) of 5 MHz were purchased from Q-Sense Corp. (Sweden). Initially, the PAM-*g*-lactose modified QCM-D resonator was washed with distilled water for three times. Then it was dried by a flow of nitrogen gas and was put into a flow-cell for frequency measurement, in which the cell temperature was maintained at 25 ℃. After stabilization of fundamental resonance frequency with pure water and monosaccharide solution (0.02 mol L^{-1}, Neu5Ac or galactose) was pumped into flow-cell by a peristaltic pump at a constant speed of 0.100 ml min^{-1}. The frequency change was recorded by Q-Sense software and analyzed by Q-Tools.

Cell culture and protein extraction

Cell culture and protein extraction were carried out as reported.[8] HeLa S3 cells were cultured in RPMI-1640 with 10% FBS, 4.5 g L^{-1} glucose, 2 mM glutamine, and 100 μg mL^{-1} penicillin/streptomycin at 37 ℃ with 5% CO_2. The native cells were harvested when they reached 90% confluence in T-75 flasks. To extract protein from the cells, the cells were centrifuged at 1000 rpm and washed with 1x phosphate buffered saline (PBS, pH: 7.4, 10 mM) twice. The resulting cells were mixed with precooled lysis buffer (50 mM tris, pH=7.4, 8 M urea, 65 mM DTT, 1 mM EDTA, 1% (v/v) protease inhibitor cocktail, 1% (v/v) Triton X-100). After sonication for three cycles, the resulting solution was transferred to centrifuge tube and centrifuged at 15,000×g for 30 minutes at 4 ℃. The supernatants were collected and stored at –80 ℃ for further use.

Trypsin digestion of proteins

Digestion of standard proteins were carried out with trypsin. Standard proteins including fetuin and BSA (1 mg) were dissolved with 6 M urea in 50 mM NH_4HCO_3 (100 μL). The disulfide bonds in proteins were disrupted with 50 mM DTT (2 μL). The resulting solution was stored at 56 ℃ for 45 minutes and 50 mM IAA (5 μL) was added. The solution was incubated in the dark for 30 minutes at room temperature. Finally Trypsin was used to digest each protein at an enzyme to protein ratio of 1:30. After incubation of trypsin and protein at 37 ℃ for 16 hours, the digestion was stopped by adding FA to the final concentration of 1%.

Mass spectrometer analysis and database search

The obtained peptide fractions from tryptic digests of standard proteins were analyzed with a nano electrospray ionization-quadrupole time-of-flight mass spectrometer (ESI Q-TOF MS, Waters, and Milford, MA, USA). The peptides were directly infused into ESI source with Nano Acquity UPLC (Waters, Milford, MA, USA). The positive MS scan was acquired at m/z 600-2000. The separation and characterization of glycopeptides from HeLa S3 cell lysate were performed with LTQ-Orbitrap Velos coupled with Accela 600 HPLC system (Thermo, San Jose, CA). For the RPLC separation, mobile phases A and B were 0.1% FA in water and 0.1% FA in

acetonitrile, respectively. The analytical column with a 75 μm i.d. was packed with C18 AQ particles (3 μm, 120 Å) to 10 cm length. The flow rate was set at 200 nL min^{-1}. The 120 min gradient elution was performed with a gradient of 2-35% B in 110 min, 35-85% B in 5min, 85% B in 5 min. Full mass scan were recorded with mass range from m/z 400 to 2000 (R = 60,000 at m/z 400). The 10 most intense ions from the full scan were selected for fragmentation via collision induced dissociation (CID) in the ion trap (relative collision energy for CID was set to 35%). The dynamic exclusion function was set as follows: repeat count 1, repeat duration 30 s, and exclusion duration of 60 s.

The RAW files collected by Xcalibur 2.1 were converted to *.MGF by Proteome Discoverer (v1.2.0.208, Thermo, San Jose, CA) and searched with Maxquant.[9] (version 1.3.0.5, Max Planck Institute of Biochemistry, Martinsried, Germany) using Uniprot protein fasta database of human (88473 entries). Cysteine carboxamidomethylation (C) was set as a fixed modification, oxidation on methionine (M) and deamidation (NQ) were set as the variable modifications. Up to two missing cleavages of trypsin were allowed. Mass tolerances were set as 10 ppm and 0.1 Da for the parent and fragment ions, respectively. A false discovery rate (FDR) of 1% was set for peptide identification.

Part S2 Supporting Tables and Figures

Table S1 Association constants (K_a) of fluorescein-labelled lactose with different monosaccharides in aqueous solutions at pH 7.4 and pH 3.8 at 20 ℃.

**Saccharides**	**K_a [× 10^3 L mol^{-1}]^{a,b}**	
	**pH=7.4**	**pH=3.8**
Galactose	14.05 ± 1.55	24.28 ± 2.68
Fucose	4.45 ± 0.37	24.81 ± 2.73
Glucose	15.26 ± 1.68	34.25 ± 3.77
GalNAc	24.46 ± 2.69	46.44 ± 5.11
mannose	5.76 ± 0.50	47.06 ± 5.18
GlcNAc	29.03 ± 3.20	52.72 ± 5.80
Neu5Ac	2.54 ± 0.25	161.51 ± 16.33

[a] K_a values were obtained from fluorescence titration experiments according to intensity changes at the emission-peak maximum.
[b] All error values were obtained by the results of nonlinear curve fitting, the correlation coefficient (R) of nonlinear curve fitting is over 0.99. The nonlinear calculation equation is

listed as below:

$$F = F_0 + \frac{F_{lim} - F_0}{2C_0}\{C_H + C_G + 1/K_a - [(C_H + C_G + 1/K_a)^2 - 4C_H C_G]^{1/2}\}$$

Where F represents the fluorescent intensity, and C_H and C_G are the corresponding concentrations of host and guest.

Table S2 Association constants (K_a) of fluorescein-labelled lactose and fluorescein-labelled acetylated lactose with different monosaccharides in aqueous solutions at pH 7.4 at 20 ℃.

Saccharides	K_a [$\times 10^3$ L mol^{-1}]	
	fluorescein-labelled lactose	fluorescein-labelled acetylated lactose
Galactose	14.05 ± 1.55	0.52 ± 0.06
Fucose	2.45 ± 0.27	1.03 ± 0.11
Glucose	15.26 ± 1.68	2.65 ± 0.29
GalNAc	24.46 ± 2.69	5.08 ± 0.56
Mannose	2.76 ± 0.30	0.90 ± 0.1
GlcNAc	29.03 ± 3.20	12.08 ± 1.33
Neu5Ac	2.54 ± 0.25	0.154 ± 0.017

Table S3 Association constants (K_a) of fluorescein-labelled saccharide hosts with Neu5Ac and glucose in aqueous solutions at pH 3.8 at 20 ℃.

Saccharide Hosts	K_a [$\times 10^3$ L mol^{-1}]	
	Neu5Ac	glucose
Lactose	161.51 ± 16.33	34.25 ± 3.77
Maltose	102.45 ± 10.27	56.34 ± 6.11
Cellobiose	95.26 ± 9.68	45.65 ± 5.29

Table S4. List of identifed glycopeptide information for the fetuin enrichment experiment.

**Position**	**Peptide sequence**	**peptide mass**	**Glycan**	**glycan mass**	**glycopeptide mass M+**	
**144-159**	KLCPDCPLLAPL N(156) DSR	1868.9357	[Hex]6[HexNAc]5 [NueAC]2	2589.5498	4440.4855	1480.8285 (3+)

**72-103**	RPTGEVYDIEIDT LETTCHVLDPTP LAN (99) CSVR	3557.7250	[Hex]4[HexNAc]5	1682.6900	5222.4150	1741.4717 (3+)		
**72-103**	RPTGEVYDIEIDT LETTCHVLDPTP LAN (99) CSVR (Cys_CM: 89, 100)	3671.7679	[Hex]4[HexNAc]3	1276.2700	4930.0379	1644.0126 (3+)		
		3671.7679	[Hex]5[HexNAc]4 [NueAC]1	1932.9099	5586.6778	1397.4195 (4+)		
		3671.7679	[Hex]5[HexNAc]4 [NueAC]2	2224.1798	5877.9477	1470.2369 (4+)	1176.3895 (5+)	
		3671.7679	[Hex]6[HexNAc]5 [NueAC]2	2589.5498	6243.3177	1561.5794 (4+)	1249.4635 (5+)	
		3671.7679	[Hex]6[HexNAc]5 [NueAC]3	2880.8197	6534.5876	1634.3969 (4+)	1307.7175 (5+)	1643.8969 (4+)
		3671.7679	[Hex]6[HexNAc]5 [NueAC]4	3172.0896	6825.8575	1707.2144 (4+)		
**160-211**	VVHAVEVALATF NAESN(176)GSY LQLVEISRAQFVP LPVSVSV EFAVAATDCIAK (Cys_CM: 208)	5459.6304	[Hex]3[HexNAc]3	1114.1100	6555.7404	1639.6851 (4+)	1311.9481 (5+)	1649.1851 (4+)
**160-187**	VVHAVEVALATF NAESN (176) GSYLQLVEISR	3016.5738	[Hex]6[HexNAc]5 [NueAC]4	3172.0896	6170.6634	1543.4159 (4+)		

Table S5. The identified glycopeptides from Hela cell lysate with PAM-*g*-lactose$_{0.11}$.

See the separate Excel file!

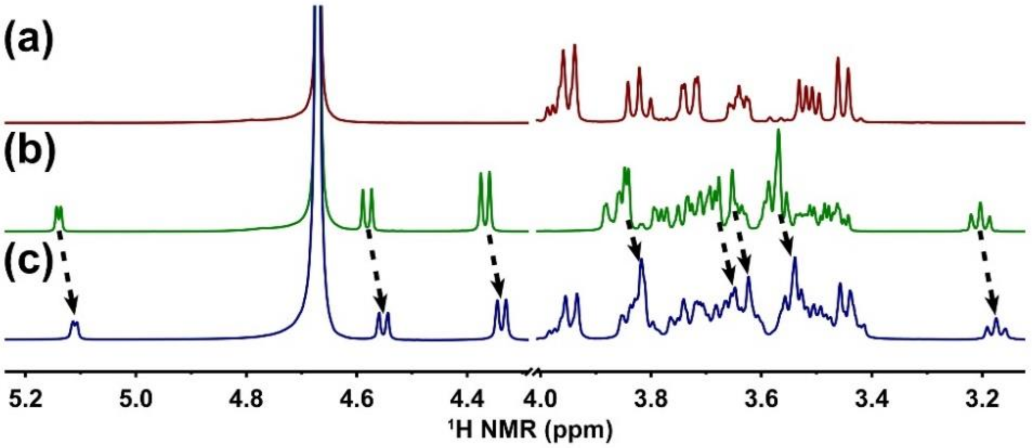

Fig. S1 ^{1}H NMR spectra of Neu5Ac (a), lactose (b) and mixture of Neu5Ac with equimolar ratio of lactose (c) in D_2O at pH 3.8 and 20 °C. Clear upfield shifts of the lactose protons are indicated by black dotted lines.

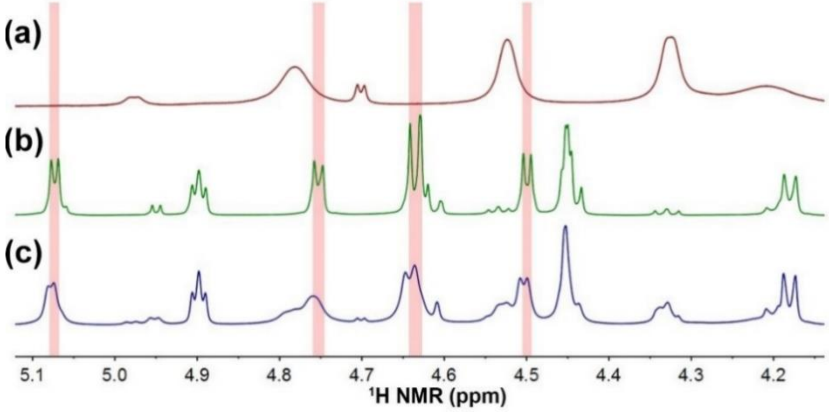

Fig. S2 ^{1}H NMR spectra of Neu5Ac (a), lactose (b) and their mixture at a molar ratio of 1:1 (c) in DMSO-d_6 at 20 ℃. Evidential changes in chemical shifts were observed for the C-H protons of lactose, revealing intensive H-bond interactions between Neu5Ac and lactose.

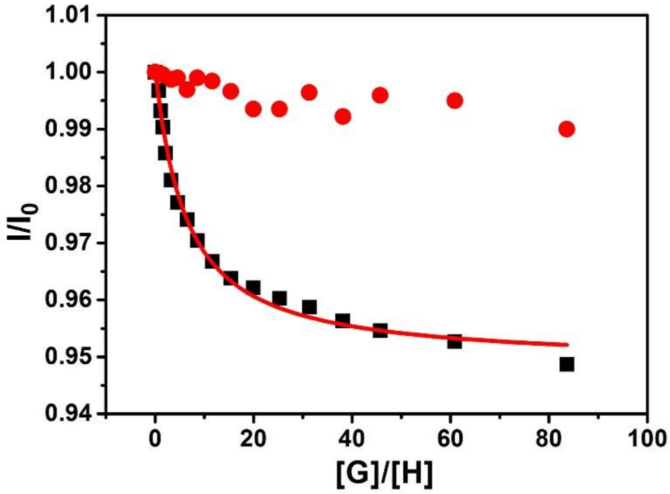

Fig. S3 Fluorescence intensity change (I/I_0) of fluorescein-labelled lactose (1.0×10^{-6} mol L^{-1}) with (red dots, 5.0×10^{-4} mol L^{-1}) and without urea (black dots) upon the addition of various equivalents of Neu5Ac in formate-buffer solution (10 mM, pH 3.8) at 20 °C. For red dots, reliable K_a value could not be obtained due to too small fluorescent intensity change.

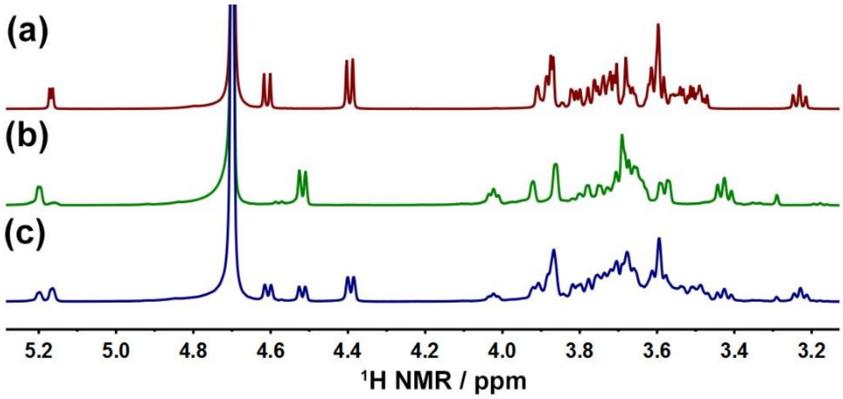

Fig. S4 ^{1}H NMR spectra of lactose (a), galactose (b) and their mixture at a molar ratio of 1:1 (c) in D_2O at pH 3.8 and 20 °C. No evidential change in chemical shifts was observed for the C-H protons of both lactose and galactose, indicating a rather weak complexation between them.

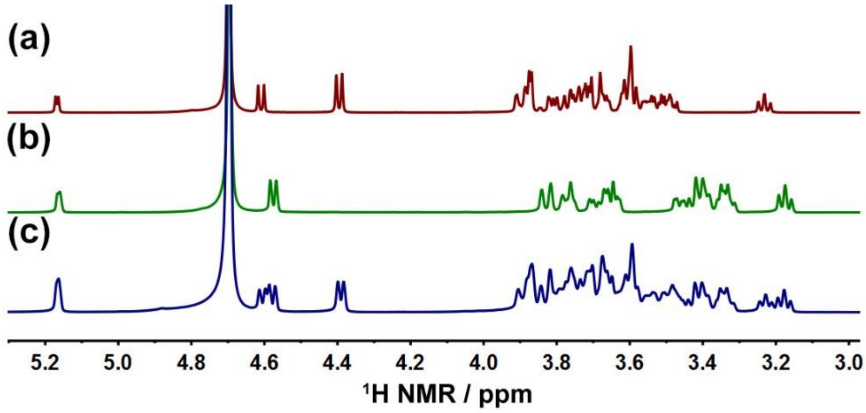

Fig. S5 ^{1}H NMR spectra of lactose (a), glucose (b) and their mixture at a molar ratio of 1:1 (c) in D_2O at pH 3.8 and 20 ^oC. No evidential change in chemical shifts was observed for the C-H protons of both lactose and glucose, indicating a rather weak complexation between them.

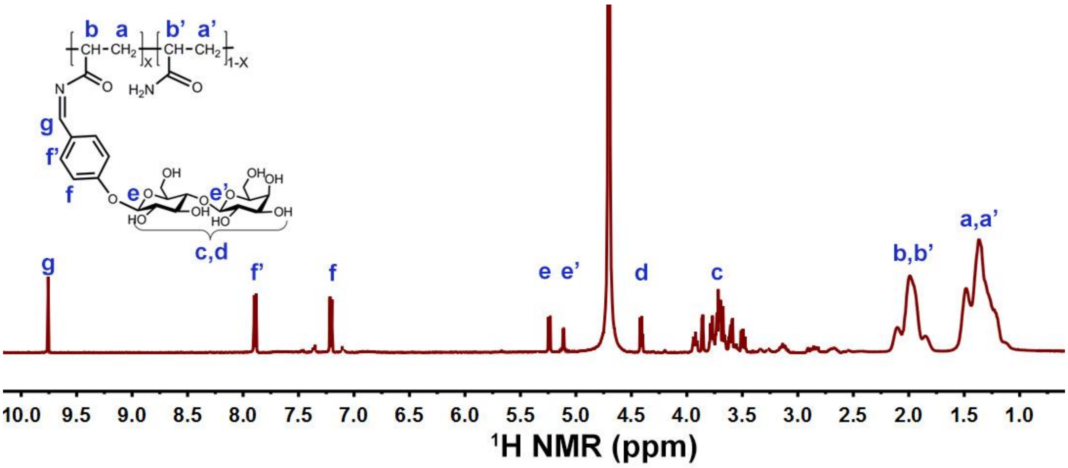

Fig. S6 ^{1}H NMR spectrum of PAM-*g*-lactose, the hydrogen proton assignments are indicated by blue letters, and the corresponding chemical structure is shown in the inset. From the spectrum, the grafting density of lactose unit in PAM-*g*-lactose was calculated to be about 11%.

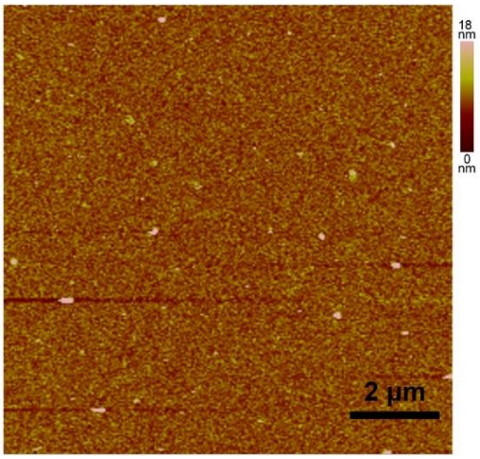

Fig. S7 The surface morphology of PAM-*g*-lactose film on silicon wafer (scan area 10×10 μm^2).

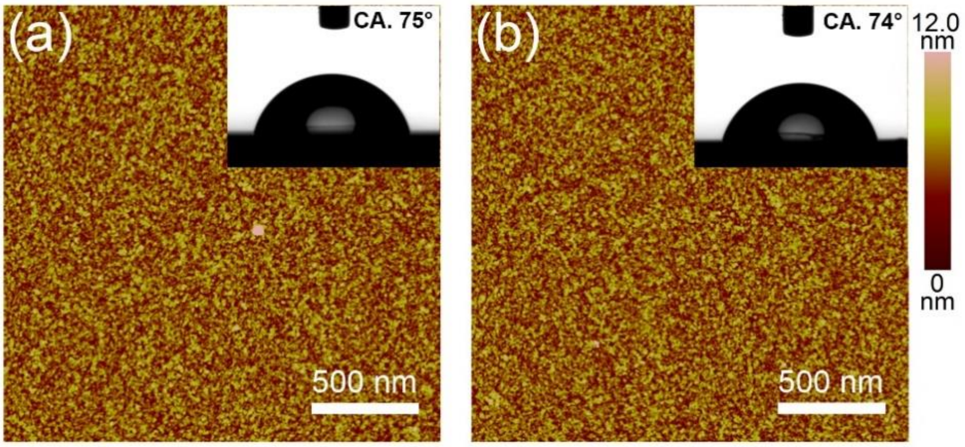

Fig. S8 The surface morphology (a, b) of PAM-*g*-lactose film on silicon wafer after being treated by the galatose (a) and glucose (b) solution (0.02 mol·L^{-1}). Inserts of (a) (b) are corresponding surface water drop profiles.

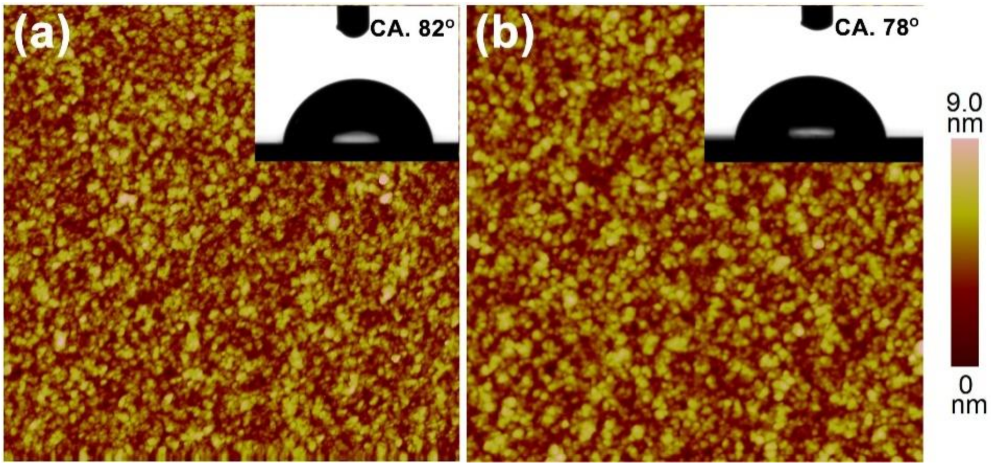

Fig. S9 The surface morphology of PAM film on silicon wafer before (a) and after (b) being treated by Neu5Ac solution (0.02 mol·L^{-1}). Inserts of (a) (b) are corresponding surface water drop profiles.

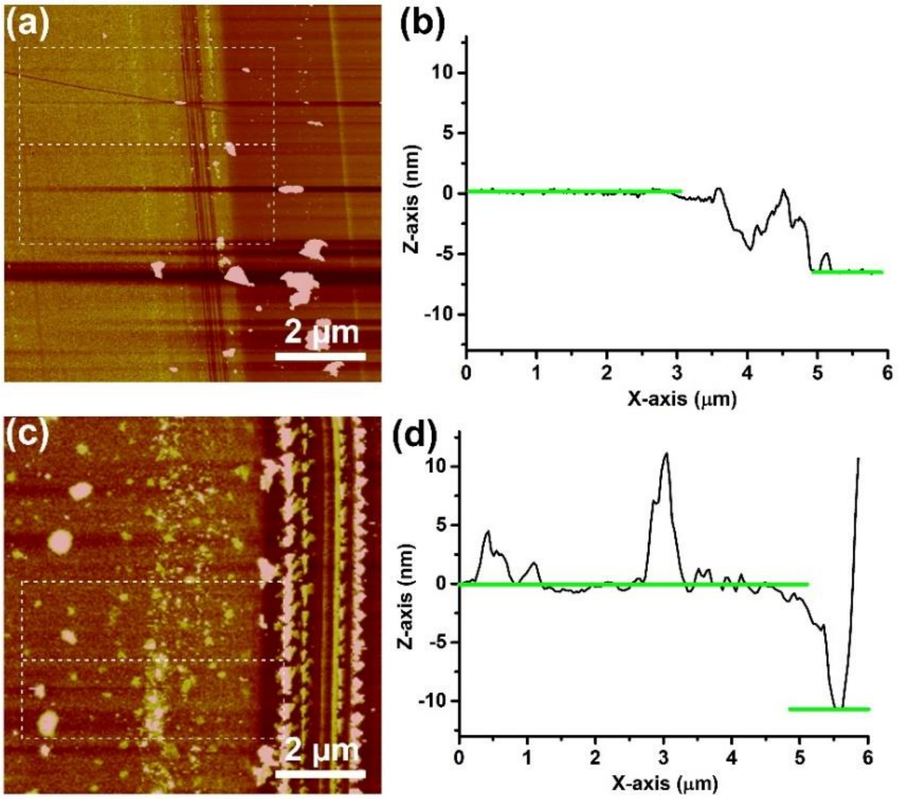

Fig. S10 AFM height images of the PAM-*g*-lactose film on flat silicon substrates before (a, b) and after (c, d) being treated by Neu5Ac. Section profile (b, d) for the corresponding AFM image (a, c) area inside the white dotted line. The thickness of the original PAM-*g*-lactose film is about 6 ± 1 nm (b). After being treated by Neu5Ac solution (0.02 mol·L^{-1}), the thickness of the PAM-*g*-lactose film becomes into 12 ± 2 nm (d).

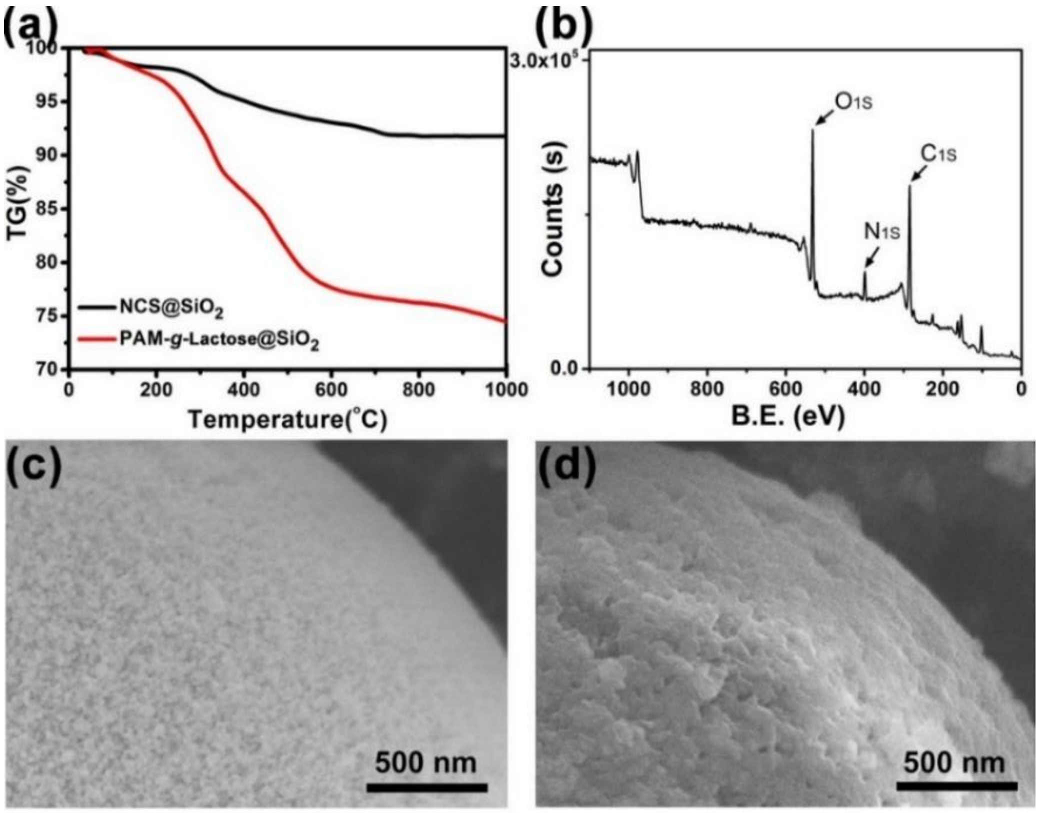

Fig. S11 (a) Thermo-gravimetric analysis (TGA) curves of isothiocyanate-activated silica gels (black) before and after modification with PAM-*g*-lactose (red). These curves demonstrated that the graft polymer was successfully immobilized on the silica gels. (b) XPS spectrum of PAM-*g*-lactose@SiO_2. SEM images of silica microspheres before (c) and after (d) being grafted with PAM-*g*-lactose. These data clearly demonstrated that PAM-*g*-lactose had been successfully immobilized on the silica microspheres. Abbreviation: PAM, polyacrylamide.

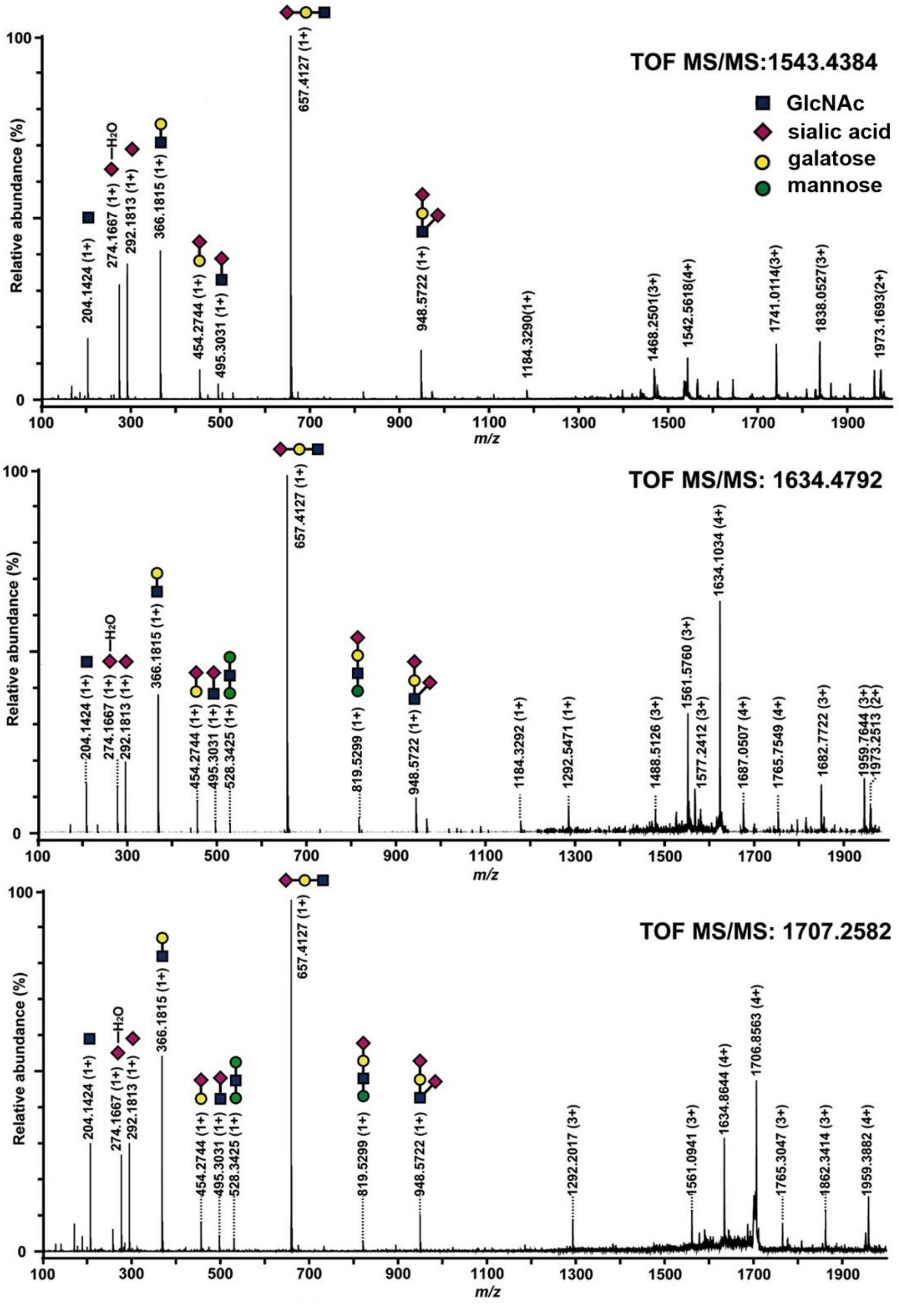

Fig. S12 Part of tandem mass spectra of glycopeptides enriched from peptide mixture of fetuin and different folds of BSA. These spectra of peptides with m/z at 1543.4384 (4+), 1634.4792 (4+), and 1707.2582 (4+) demonstrated the existence of sialic acid residues.

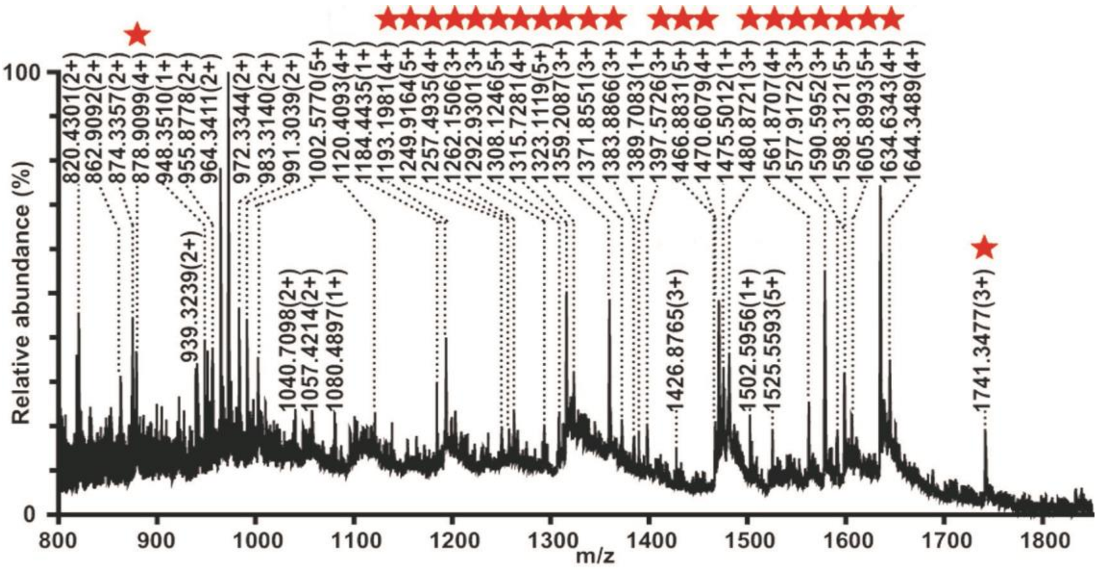

Fig. S13 Mass spectra of glycopeptides enriched polyacrylamide@SiO_2 (PAM@SiO_2) from the tryptic digests of fetuin and BSA at a molar ratio of 1:10. Glycopeptides are labelled with red pentacles; and the non-glycopeptides are only marked with their m/z values. The PAM@SiO_2 material could not work when the BSA interference level was higher than 10-fold fetuin, implying a poor enrichment selectivity.

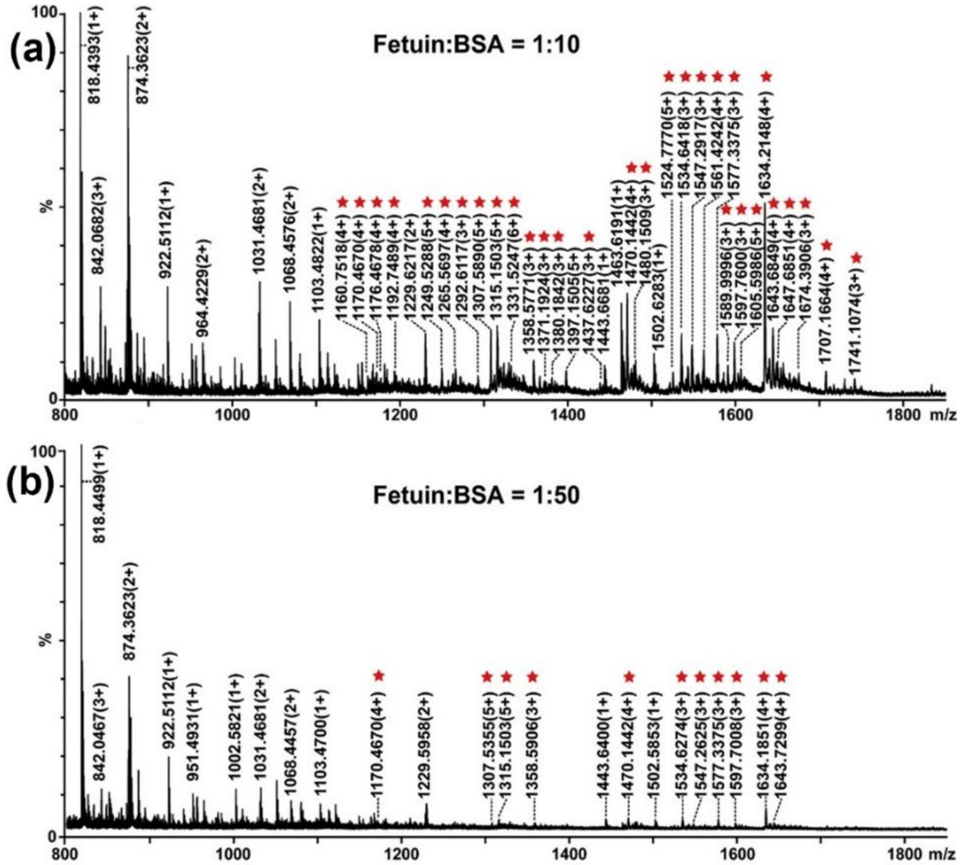

Fig. S14 Mass spectra of glycopeptides enriched by Sepharose from the tryptic digests of fetuin and 10-fold (a) or 50-fold (b) BSA. The non-glycopeptides are only marked with their m/z

values and glycopeptides are marked with red pentacles. As the best commercial materials applied in glycoproteomics, the enrichment selectivity of Sepharose is not satisfied, which could not work well when the BSA interference level is higher than 50-fold fetuin.

References

[1] Bejugam, M & Flitsch, S. L. An efficient synthetic route to glycoamino acid building blocks for glycopeptide synthesis. *Org. Lett.* **6**, 4001-4004 (2004).

[2] Zheng, Y. *et al*. Synthesis and anti-tumor activities of 5-carbohydrate modified cyclophosphamide derivates. *J. Chin. Pharm. Sci.* **19**, 327-340 (2010).

[3] Wen, H. et al. Synthesis and biological evaluation of helicid analogues as novel acetylcholinesterase inhibitors. *Eur. J. Med. Chem.* **43**,166-173 (2008).

[4] Uzgören Baran, A. Comparative study of microwave-assisted and conventional synthesis of ibuprofen-based acyl hydrazone derivatives. *Turk. J. Chem.* **37**, 927-935 (2013).

[5] Custelcean, R., Gorbunova, M. G. & Bonnesen, P. V. Steric Control over Hydrogen Bonding in Crystalline Organic Solids: A Structural Study of *N,N*'-Dialkylthioureas. *Chem. -Eur. J.* **11**, 1459-1466 (2005).

[6] Gradinaru, C. C., Marushchak, D. O., Samim, M. & Krull, U. J. Fluorescence anisotropy: from single molecules to live cells. *Analyst* **135**, 452-459 (2010).

[7] Derjaguin, B. V., Muller, V. M. & Toropov, Y. P. Effect of contact deformations on the adhesion of particles. *J. Colloid Interface Sci.* **53**, 314-326 (1975).

[8] Kaji, H., Yamauchi, Y., Takahashi, N. & Isobe, T. Mass spectrometric identification of *N*-linked glycopeptides using lectin-mediated affinity capture and glycosylation site-specific stable isotope tagging. *Nat. Protoc*. **1**, 3019-3027 (2006).

[9] Mysling, S., Palmisano, G., Højrup, P. & Thaysen-Andersen, M. Utilizing Ion-Pairing Hydrophilic Interaction Chromatography Solid Phase Extraction for Efficient Glycopeptide Enrichment in Glycoproteomics. *Anal. Chem.* **82**, 5598-5609 (2010).
